# Plasmonic Silver‐Nanoparticle‐Catalysed Hydrogen Abstraction from the C(*sp* ^*3*^)−H Bond of the Benzylic C_α_ atom for Cleavage of Alkyl Aryl Ether Bonds

**DOI:** 10.1002/anie.202215201

**Published:** 2022-12-16

**Authors:** Pengfei Han, Xin Mao, Yichao Jin, Sarina Sarina, Jianfeng Jia, Eric R. Waclawik, Aijun Du, Steven E. Bottle, Jin‐Cai Zhao, Huai‐Yong Zhu

**Affiliations:** ^1^ College of Chemistry and Chemical Engineering Hunan University Changsha 410082 P. R. China; ^2^ School of Chemistry and Physics Queensland University of Technology Brisbane QLD 4001 Australia; ^3^ School of Chemical and Material Science Shanxi Normal University Linfen 041000 P. R. China; ^4^ Beijing National Laboratory for Molecular Sciences Institute of Chemistry Chinese Academy of Sciences Beijing 100190 China

**Keywords:** C(sp^3^)−H Bond Activation, Ether Bond Cleavage, Plasmonic Catalysis, Product Selectivity, *β*-O-4 Linkage

## Abstract

Selective activation of the C(sp^3^)−H bond is an important process in organic synthesis, where efficiently activating a specific C(sp^3^)−H bond without causing side reactions remains one of chemistry's great challenges. Here we report that illuminated plasmonic silver metal nanoparticles (NPs) can abstract hydrogen from the C(sp^3^)−H bond of the C_α_ atom of an alkyl aryl ether *β*‐O‐4 linkage. The intense electromagnetic near‐field generated at the illuminated plasmonic NPs promotes chemisorption of the *β*‐O‐4 compound and the transfer of photo‐generated hot electrons from the NPs to the adsorbed molecules leads to hydrogen abstraction and direct cleavage of the unreactive ether C_β_−O bond under moderate reaction conditions (≈90 °C). The plasmon‐driven process has certain exceptional features: enabling hydrogen abstraction from a specific C(sp^3^)−H bond, along with precise scission of the targeted C−O bond to form aromatic compounds containing unsaturated, substituted groups in excellent yields.

## Graphical abstract

The intense electromagnetic near‐field and hot electrons of illuminated plasmonic metal nanoparticles enable hydrogen abstraction from the C(sp^3^)−H bond of the Cα atom of β‐O‐4 linkages. The cleavage of unreactive ether C_β_−O bonds is facilitated to produce aromatic compounds with unsaturated double bonds.

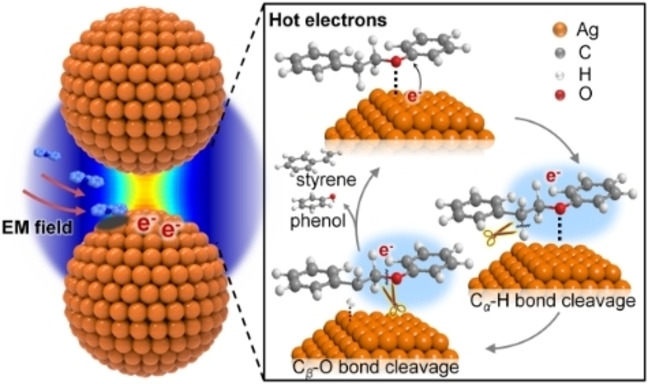

## Introduction

Plasmonic metal (gold, silver, copper or aluminium) NPs have emerged over the last decade as a new class of photocatalysts that utilise visible light.^[1]^ When irradiated by visible light, plasmonic metal NPs can efficiently absorb photons due to the localised surface plasmon resonance (LSPR) effect, whereby the conduction electrons of the NPs collectively oscillate in resonance with the electromagnetic field of incident light.^[2]^ The LSPR absorption can generate energetic hot charges (electrons and holes),[^1h^, ^3^] and intense electromagnetic (EM) near‐fields around the nanostructures,[^1g^, ^3a^] and significant localised heating.^[4]^ The LSPR effect has been used to initiate catalysis on NP surfaces under mild reaction conditions.^[3]^ In contrast to semiconductor photocatalysis, LSPR light adsorption can promote reactions not only by the well‐known hot charge carrier‐mediated chemical conversion[^1b^, ^1c^, ^1d^, ^5^] but also through other novel mechanisms. Acting as efficient antennas for light, plasmonic NPs can pass energy or hot charges to catalytically active sites of transition metal atoms directly alloyed to the NP surface,[^3c^, ^6^] or to metal ions near the NPs via molecular bridges.^[7]^ The NP antennas can also effectively concentrate incident light to high intensity in narrow gaps between the NPs, “hot spots”,^[8]^ and significantly enhance acid catalysis in these regions.^[9]^ Additionally, the NPs can activate the reactant adsorbed on the NPs for reactions via resonance energy transfer.^[10]^ The sharp electromagnetic‐field gradient of the illuminated plasmon metal NPs generates an EM intensity gradient force^[11]^ that enhances the adsorption of some light‐polarisable reactants on the catalyst, which has been shown to switch reaction pathways in some instances.^[12]^ Recently, we found LSPR enhanced transmetalation from copper NPs to palladium NPs, enabling control over cross‐coupling and homo‐coupling reaction pathways by tuning light irradiation wavelength.^[13]^ These studies demonstrate that new plasmonic photocatalyst systems can access novel and often greener, reactive channels not achievable by either conventional heating or semiconductor photocatalysis. Using plasmonic catalysts, it is possible to develop entirely new approaches to achieve chemical synthesis.

For example, selective activation of the C(*sp* ^3^)−H bond is critical in organic synthesis, since it opens up a range of useful synthetic routes. However, efficiently activating a specific C(*sp* ^3^)−H bond without causing side reactions remains a formidable challenge because of the high C(*sp* ^3^)−H bond energy.^[14]^ Pd‐based catalysts have been employed to activate C(*sp* ^3^)−H bonds with a typical bond dissociation energy (BDE) at about 364 kJ mol^−1^.^[15]^ Other considerations include reactivity towards other saturated bonds and general catalyst stability and cost. Therefore, activating the benzylic C_α_−H bond over non‐Pd catalysts under mild conditions is of great interest. The interaction of hydrogen with Ag is much weaker than with other noble metals, Au, Pd and Pt.^[16]^ The visible‐light absorption of Ag NPs is attributed to the LSPR effect, while the light absorption of Au, Pd and Pt includes direct interband excitation.^[17]^ We focus on Ag NPs‐based plasmonic catalysts in the present study for two reasons. First, the intense LSPR absorption of short wavelengths (peaked around 400 nm) by Ag NPs efficiently gains the energy that can overcome higher activation energy barriers. Second, the light absorption is predominately due to the LSPR effect. It provides an opportunity to demonstrate that plasmonic catalysts can access new mechanisms and might achieve previously challenging transformations.

## Results and Discussion

Using density functional theory (DFT), we analysed BDEs of the benzylic C_α_−H and C_β_−H bonds of 1‐phenoxy‐2‐phenylethane, a molecule with the *β*‐O‐4 linkage that represents up to 60 % of the linkages in lignin.^[18]^ The results show that the BDE of the benzylic C_α_−H bond is as high as 470 kJ mol^−1^ (Entry 1 in Table S1, Supporting Information) and cleaving such bonds under mild reaction conditions cannot therefore occur. However, the BDEs of C_α_−H and C_β_−H bonds in the [C_14_H_14_O]^−^ transient negative ion (TNI) are 250 kJ mol^−1^ and 329 kJ mol^−1^ (Entries 2 and 3, Table S1), respectively. The hot electron transfer to the molecule could substantially lower the energy requirement of H‐abstraction from C_α_−H. It reveals an opportunity to selectively active C_α_−H using illuminated plasmonic metal NPs.

**Figure 1:**
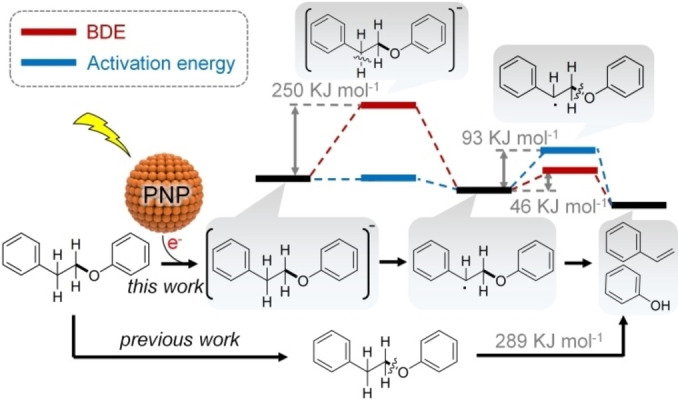
Calculated activation energy of TNI, dissociation energy of C_α_−H bonds and alkyl ether C−O bonds in 1‐phenoxy‐2‐phenylethane molecule after hot electrons transferred from illuminated plasmonic nanoparticle (PNP).

Moreover, the DFT calculations indicate that the formation of the C_α_ radical considerably lowers the BDE of the C_β_−O bond and the activation energy barrier of target products to 46 kJ mol^−1^ and 93 kJ mol^−1^ (Figure 1 and Entry 4 in Table S1), respectively. It gives an energy‐optimised reaction route for cleavage of the ether bond, which favourably compares with direct C_β_−O bond cleavage (with a BDE of 289 kJ mol^−1^, Entry 5 in Table S1) or C_β_ radical formation pathway (with a BDE of 371 kJ mol^−1^, Entry 6 in Table S1).

Precise scission of targeted alkyl ether (C−O) bonds with others remaining intact is a challenge and of great interest. The alkyl ether bonds are relatively unreactive, and they are only slightly weaker than the C(*sp* ^3^)−C(*sp* ^3^) bonds (289 kJ mol^−1^ vs 304.8 kJ mol^−1^).^[19]^ Various approaches have been proposed.^[20]^ A creative approach is oxidising the C_α_ from C_α_−H or C_α_−OH to C_α_=O, which substantially facilitates the cleavage of the robust ether C−O bonds.[^20a^, ^20b^]

Based on the above analysis, we have proposed that if the light absorption of plasmonic NPs could excite hot electrons and the hot electrons could transfer to the molecule with the ether (C−O) bonds, there would be new reaction pathways for the H‐abstraction from benzylic C_α_−H bonds and the cleavage of the robust ether C_β_−O bonds. For the reaction pathway via the plasmon‐mediated cleavage of the *sp* ^3^ hybridised C_α_−H bond, cleaving the C_α_−H bond with a BDE of 250 kJ mol^−1^ (Figure 1) has the highest energy barrier. The electrons excited by illuminating plasmonic Ag NPs with visible light could provide energy to overcome the barrier.

To verify the plasmon‐mediated H‐abstraction and ether C_β_−O bond cleavage, we conducted cleavage of 1‐phenoxy‐2‐ phenylethane, a model molecule with *β*‐O‐4 linkage, over plasmonic NP catalysts under visible‐light irradiation. The details of catalyst preparation and photocatalytic reaction are provided in Supporting Information. The model compound can be catalytically cleaved at a temperature range between 100 °C and 120 °C in the dark under high H_2_ pressures, yielding phenol and ethylbenzene.^[18]^

**Figure 2:**
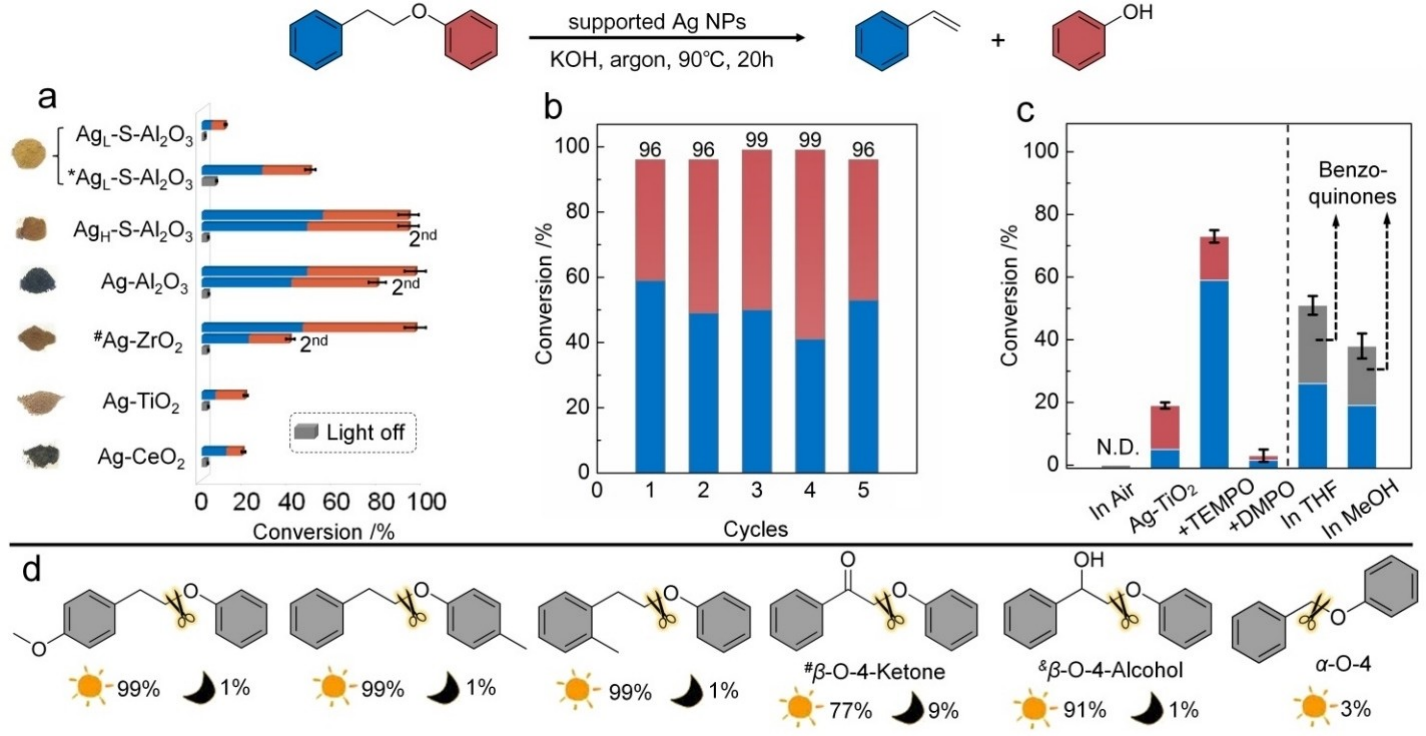
a) Catalytic performances of the supported Ag NPs for C−O bond cleavage of 1‐phenoxy‐2‐phenylethane under visible‐light irradiation and in the dark. Reaction conditions: 1‐phenoxy‐2‐phenylethane (*β*‐O‐4 model, 0.05 mmol), KOH (0.15 mmol), 2 mL of isopropanol solvent, 1 atm Ar atmosphere, 10 mg of catalysts, under irradiation of 0.5 W cm^−2^ from a LED lamp of 440 nm wavelength for 20 h at 90±2 °C. *33 mg of a catalyst was added. The Insets show photographs of the as‐prepared catalysts. Their DR UV/Vis spectra are provided in Figure S1. b) Recyclability of Ag_H_‐S‐Al_2_O_3_ photocatalysts for 1‐phenoxy‐2‐phenylethane conversion. c) Influence of the atmosphere, support, additive, and solvent on the photocatalytic performance of 10 mg Ag NP‐based catalysts. Unless specified, other conditions were the same as those for panel a. d) Catalytic performances of Ag_H_‐S‐Al_2_O_3_ photocatalyst for the cleavage of different *β*‐O‐4 model compounds. ^#^Under the irradiation of 0.2 W cm^−2^ from a LED lamp of 410 nm wavelength at 80±2 °C. ^&^Under the irradiation of 0.5 W cm^−2^ from a LED lamp of 440 nm wavelength at 50±2 °C, Other conditions were the same as those for panel a unless specified. In panels a‐c, blue bars indicate the selectivity of styrene, and red bars the selectivity of phenol. In panel c, grey bars indicate the selectivity of benzoquinones.

We found that visible‐light illumination can drive *β*‐O‐4 linkage cleavage over supported Ag NPs to yield styrene and phenol products. The results of supported Ag NPs are summarised in Figure 2. It is the first time that unsaturated products such as styrene are observed as the main product of reductive cleavage of alkyl aryl ether C−O bonds (Figure 2a–c). We also examined three more compounds with a *β*‐O‐4 linkage containing C_*α*_−H and C_*β*_−H (Figure 2d). A similar light‐induced selective cleavage of the C−O bond can occur over the Ag_H_‐S‐Al_2_O_3_ catalyst, producing only styrene and phenol derivatives. Moreover, when the H at the C_*α*_ atom is substituted by an ‐OH group or the C_*α*_ is oxidised to a carbonyl group, cleaving off the *β*‐O‐4 linkage is more readily achieved (Figure 2d). This indicates that a new mechanism for the bond scission is occurring in this system.

A range of Ag NPs catalysts on different supports were prepared by an impregnation‐reduction method, and quantitative silver contents were confirmed by inductively coupled plasma mass spectrometry (ICP‐MS, Table S2). The catalytic performances of the supported Ag NPs in the dark and under visible‐light irradiation were first investigated. As seen in Figure 2a, visible‐light irradiation is required to effectively drive the C−O bond cleavage of 1‐phenoxy‐2‐phenylethane. The use of inert photocatalytic supports (S‐Al_2_O_3_, Al_2_O_3_ and ZrO_2_) to immobilise Ag NPs results in superior activities for the cleavage of *β*‐O‐4 linkages under irradiation. The conversion of 1‐phenoxy‐2‐phenylethane over Ag_H_‐S‐Al_2_O_3_ photocatalyst under a light emission diode (LED) lamp of 440 nm wavelength and 0.5 W cm^−2^ of intensity is the highest, 96 % (Figure 2a), while the major products are styrene and phenol. In this catalyst Ag NPs (4.3 wt % of the catalyst) were immobilised on Al_2_O_3_ nanofibres grafted with a silane that contained an amino group. Al_2_O_3_ nanofibres have large specific surface areas (≈200 m^2^ g^−1^),^[7]^ enabling many silanes with amino functional groups to be grafted. The Ag NPs firmly attach to the Al_2_O_3_ support by the amino group and exhibited superior photocatalytic performance. We investigated the catalytic durability of the best‐performing catalysts (Ag_H_‐S‐Al_2_O_3_, Ag‐Al_2_O_3_ and Ag‐ZrO_2_) via recycling experiments. The results indicate that the Ag_H_‐S‐Al_2_O_3_ catalyst maintained the highest stability for *β*‐O‐4 compound conversion in the second cycle and could be reused for five cycles without noticeable activity loss (Figure 2b). Also, the cage‐like nanofibres configuration could readily confine the NPs formed within the structure and allow reactant molecules to diffuse to the metal NPs through inter‐fibre voids. These properties bring about the best conversion efficiency and recyclability of the catalyst.

**Figure 3:**
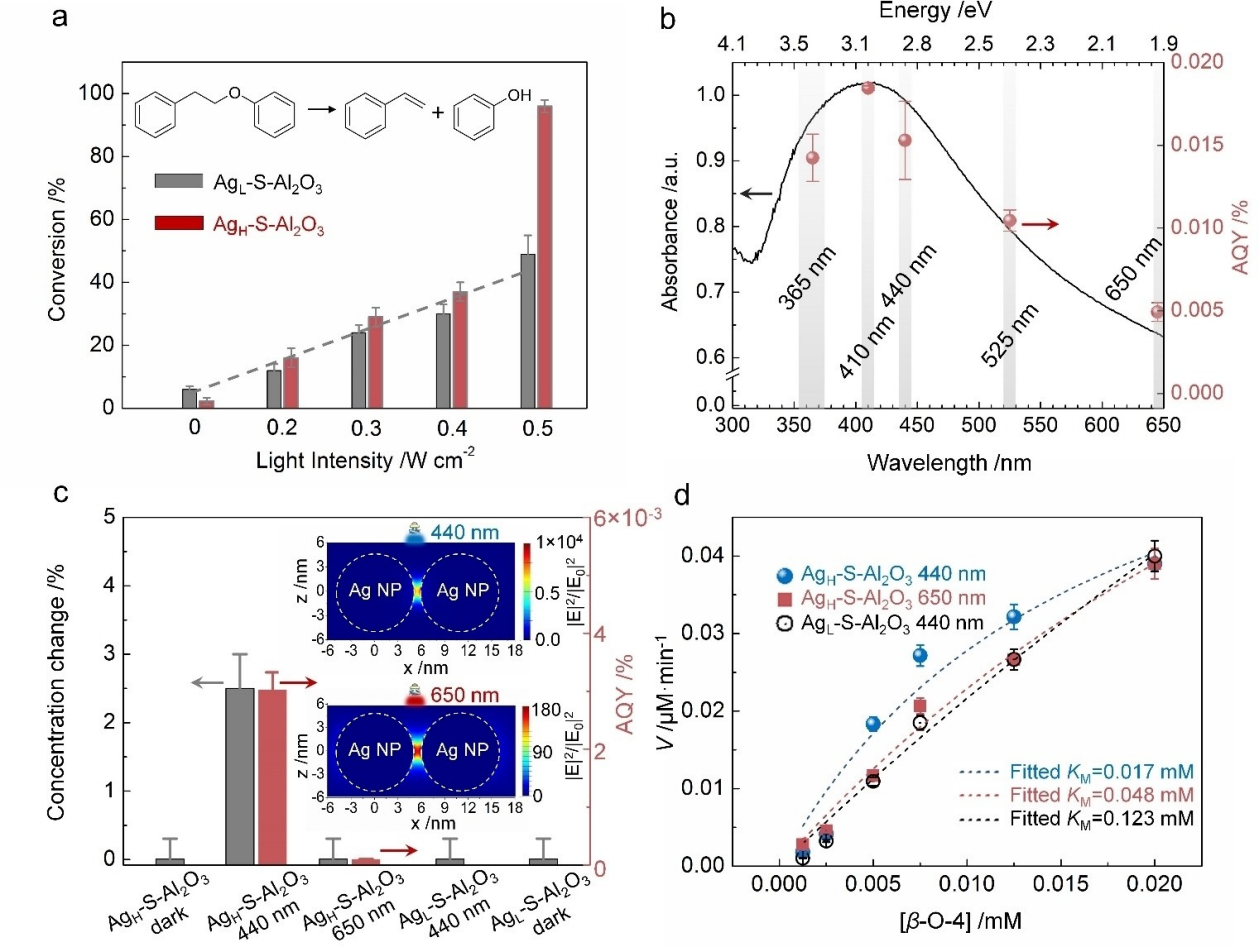
a) Dependence of the photocatalytic activities of Ag_H_‐S‐Al_2_O_3_ and Ag_L_‐S‐Al_2_O_3_ catalysts on the light irradiation intensity. The reaction conditions were the same as in Figure 2a, except the light source was a LED chip (440±5 nm) with controllable intensities from 0.2 to 0.5 W cm^−2^. b) UV/Vis spectrum of the Ag_H_‐S‐Al_2_O_3_ catalyst and dependence of its catalytic activity for cleavage of *β*‐O‐4 linkage on the light irradiation wavelength. The light intensity was 0.2 W cm^−2^ for all wavelengths. Error bars associated with conversion and AQY (y‐direction) are the standard error of three sets of unique measurements. The darker area represents the peak bandwidth of the LED. Ultraviolet (UV) and light emission diode (LED) lamps with four peak wavelengths (365±10 nm, 410±5 nm, 440±5 nm, 525±5 nm, 650±5 nm) were used to drive the reaction. c) Reactant concentration change is caused by adsorption on catalysts under irradiation and in the dark. In a typical procedure, 10 mg of Ag_H_‐S‐Al_2_O_3_ or 33 mg of Ag_L_‐S‐Al_2_O_3_ catalysts was added to 2 mL of *β*‐O‐4/2‐butanol (5×10^−3^ M) solution, filled with argon and sealed. The adsorption was conducted at 90 °C for 8 h, the same temperatures designated for the reaction. A 440 nm or 650 nm LED light at 0.5 W cm^−2^ was used as the light source. Only the *β*‐O‐4 compound and 2‐butanol can be detected by GC, for KOH is absent in the experiment. The inset shows the simulation of the near‐field enhancement between dimer Ag NPs (10 nm) with a one‐nm gap under different excitation wavelengths. The red bars represent the AQY obtained using 10 mg of Ag_H_‐S‐Al_2_O_3_ catalyst under a low reactant concentration (5×10^−3^ M) and high light intensity (0.5 W cm^−2^). d) Rates of *β*‐O‐4 compound conversion by supported Ag NPs catalysts using various reactant concentrations under monochromatic light irradiation. The Michaelis–Menten model was used to fit the experimental data to obtain the Michaelis constant (*K*_M_). The conversion data was collected after the reaction was conducted at 90 °C for 1 h, and a 440 nm or 650 nm LED light at 0.5 W cm^−2^ was used as the light source.

For the catalytic reaction on the metal particle surface, the larger specific surface area of the metal particles provides more catalytically active sites assuring a sufficiently high reaction rate. According to our previous studies,[^1c^, ^6^, ^7^] the catalytic activity decreases when the metal particle size is larger than 10 nm, and more metal particles peel off from the catalyst during the reaction. This means that poor catalyst recycling performance was observed. The Ag NPs in the Ag_H_‐S‐Al_2_O_3_ sample have the optimal sizes to exhibit intensive light absorption (see Figure 3b) and have sufficient surface sites for the reaction.

KOH was added as a typical base to promote the hydrogen donation from isopropanol (IPA) and generate a reductive/hydrogen‐rich environment. KOH favours hydrogen extraction in traditional thermocatalysis, which may benefit the H‐abstraction in the plasmonic catalysis.

An important finding is that the conversion for benzyl phenyl ether, a compound with an α‐O‐4 linkage, over the Ag_H_‐S‐Al_2_O_3_ catalyst is negligible under the same reaction conditions (Figure 2d), although the aliphatic ether bond of the α‐O‐4 compound is weaker than that of the *β*‐O‐4 linkage by 71 kJ mol^−1^.^[18b]^ If light‐induced heating, a photothermal effect, had driven the cleavage reactions, the conversion of the molecule with an α‐O‐4 linkage should be higher than that with a *β*‐O‐4 linkage. The conversion results indicate that the cleavage of the aryl alkyl linkages is not driven by thermal or photothermal effects. This is a different feature from the light‐induced heating effect in other systems.^[21]^ The plasmonic catalysis mechanism we observe appears to be novel and unique.

An interesting observation is that the conversion achieved by the Ag_H_‐S‐Al_2_O_3_ catalyst (≈96 %) was substantially higher than by the Ag_L_‐S‐Al_2_O_3_ catalyst that was prepared using the same approach as the Ag_H_‐S‐Al_2_O_3_ catalyst, but with a Ag NP content of 1.3 wt % even after increasing the added amount of the latter to 3.3 times (from 10 mg to 33 mg, Figure 2a). The silver amounts in the two catalytic systems (10 mg of Ag_H_‐S‐Al_2_O_3_ and 33 mg of Ag_L_‐S‐Al_2_O_3_) are the same (calculated from Table S2). Ag NPs in the Ag_H_‐S‐Al_2_O_3_ catalyst are slightly larger than in the Ag_L_‐S‐Al_2_O_3_ catalyst. Thus, the silver surface area in 10 mg of Ag_H_‐S‐Al_2_O_3_ is smaller than that in 33 mg of Ag_L_‐S‐Al_2_O_3_). The silver surface should be the active photocatalytic site for the reaction not proceeding on S‐Al_2_O_3_ support. To test whether this is the case, an Ag_H_‐S‐Al_2_O_3_ catalyst coated with an ultrathin silica film (SiO_2_@Ag_H_‐S‐Al_2_O_3_) was prepared (Figure S2). The 1‐phenoxy‐2‐phenylethane conversion using this SiO_2_@Ag_H_‐S‐Al_2_O_3_ catalyst was lower than 1 % under the same conditions that were used to obtain the results displayed in Figure 2a. This observation provides more evidence that the active reaction site is on the Ag NPs. We noted that the number‐density of Ag NPs is much higher in the Ag_H_‐S‐Al_2_O_3_ catalyst (Figure S3). In this sample, the distances between Ag NPs are shorter. The number‐density and size of Ag NPs significantly impact the plasmonic catalysed cleavage of the ether bond of the compounds with a *β*‐O‐4 linkage. Au‐S‐Al_2_O_3_ catalysts with different gold NP contents exhibit a similar mechanism and yield styrene and phenol as the main products (Figure S4).

When the reaction is performed in tetrahydrofuran (THF) and methanol (MeOH) solvent instead of isopropanol, both the conversion of the model compound and selectivity for phenol decrease significantly (Figure 2c). Figure S5a shows that the transparent colourless product turned dark red after the reaction in methanol, suggesting benzoquinone formation (Figure S5b and Figure S6) in the system. These observations indicate that the reactant conversion and product selectivity of the plasmon‐driven *β*‐O‐4 linkage cleavage are solvent‐dependent. When using methanol solvent, we obtained a compound with unsaturated C=O bonds. These facts indicate that the reaction over the plasmonic NP catalysts should be proceeding by a different mechanism than for thermal or photocatalytic reactions reported previously.[^7^, ^18^] Two alcohols with different polarities were mixed with isopropanol (1 : 1 v/v), respectively, as the solvent to investigate the influence of solvent polarity on the conversion of the *β*‐O‐4 model compound using Ag_H_‐S‐Al_2_O_3_ catalyst. The result shown in Table S3 indicates that both higher and lower polarity solvents led to decreased reactant conversion (Entries 1 and 2). However, the reaction can maintain a good conversion with partial replacement of IPA by 2‐butanol (Entry 3 in Table S3). According to the previous report,^[22]^ isopropanol and 2‐butanol have a similar reduction potential, much lower than methanol and capryl alcohol. Thus, the ability of the alcohol to provide H species on the Ag NPs affects the activity more significantly.

We noted that external thermal input was still necessary to achieve a high conversion of the *β*‐O‐4 compound, although plasmonic NPs can significantly lower the energy barrier for cleavage reaction. The LSPR absorption peak of Ag NPs in the Ag_H_‐S‐Al_2_O_3_ catalyst is at 410 nm wavelength. The photon energy of this wavelength is 3.02 eV. The initially excited electrons rapidly redistribute energy by electron‐electron collisions.^[23]^ Thus, most hot electrons transferred to the compound after the redistribution carried less energy, insufficient to induce the H‐abstraction. Moderate heating significantly increased the number of hot electrons with sufficient energy to overcome the activation barrier, achieving better performance. Heating also enhances mass transfer and product (e.g., styrene) desorption, facilitating reactant access to the catalytic surface.

Figures 3a and 3b show the dependence of the catalytic performance on the irradiation intensity and wavelength, respectively. Increases in the irradiation intensity result in substantial increases in *β*‐O‐4 compound cleavage. The reaction conducted in the dark shows a poor conversion (first two columns in Figure 3a). The control experiment at 120 °C in the dark indicates that an increase of 30 °C in the reaction temperature failed to enhance the Ag_H_‐S‐Al_2_O_3_ catalyst's activity (only 3 % of conversion was achieved). This corroborates that the cleavage of the aryl alkyl linkage is not driven by the photothermal effect. Besides, while keeping the light intensities identical (0.2 W cm^−2^), the efficiency of the reaction over Ag_H_‐S‐Al_2_O_3_ catalyst under 410 nm is about 4 times higher than that under 650 nm (Figure 3d). Moreover, the temperature on the surface of plasmonic NPs suspended in stirred liquid (probably only a few K above the liquid temperature) will be much lower than at the interface with gas due to the high value of the thermal conductivity of the solvent compared to the gas.^[24]^ Therefore, the electric field enhancement and hot charge carriers transfer made the dominant contribution to the catalysis rather than the light‐induced heating.[^21a^, ^21b^, ^21d^]

We observed a linear increase in *β*‐O‐4 compound conversion by Ag_L_‐S‐Al_2_O_3_ catalyst when increasing the irradiation intensity (indicated by the dashed line). This feature suggests that the cleavage is an electron‐driven reaction on metals.^[1b]^ However, the conversion is not linearly dependent on the light intensity above a threshold light intensity when using the Ag_H_‐S‐Al_2_O_3_ catalyst (Figure 3a). Above the threshold, light irradiation may enhance processes other in addition to the generation of excited hot electrons.^[7]^

We measured the adsorption capacity of the catalysts under various conditions using a method reported previously^[7]^ and found that the light‐induced chemisorption of 1‐phenoxy‐2‐phenylethane on 10 mg Ag_H_‐S‐Al_2_O_3_ catalyst is much greater than that on 33 mg Ag_L_‐S‐Al_2_O_3_ catalyst (Figure 3c). This is consistent with the result that 10 mg of Ag_H_‐S‐Al_2_O_3_ catalyst has higher activity than 33 mg of Ag_L_‐S‐Al_2_O_3_ catalyst under 440 nm wavelength light (Figure 2a). The blank experimental comparisons show that no obvious concentration change can be detected with the increases in the support amount (Table S4). So Ag NPs contributed to the adsorption, and the chemisorbed 1‐phenoxy‐2‐phenylethane molecules did not desorb from the catalyst when the light was turned off. The light‐promoted chemisorption is a reason for the non‐linear increases of 1‐phenoxy‐2‐phenylethane conversion with the rising irradiation intensity.

The Ag_H_‐S‐Al_2_O_3_ has a much higher number‐density and slightly larger Ag NPs (Figure S3). The EM near‐field around the NPs in the higher number‐density environment is more intense.[^1g^, ^3a^] We simulated the near‐field enhancement of dimer Ag NPs (the diameter is 10 nm) with a one nm gap between them using the FDTD method. Under 440 nm light excitation, the intensity of EM fields at the surface of Ag NPs is 55 times larger than that under 650 nm‐wavelength light (Figure 3c inset). Correlating the simulation results with the adsorption results of Figure 3c, we conclude that the intense near EM fields generated by closely‐packed Ag NPs promote 1‐phenoxy‐2‐phenylethane chemisorption. The larger Ag NP sizes also contribute to enhancing the near EM field.^[1l]^

The action spectrum in Figure 3b shows the effect of irradiation wavelength on the conversion over the Ag_H_‐S‐Al_2_O_3_ catalyst. The apparent quantum yields (AQY) were calculated by normalising the plasmonic catalysed conversion of reactant to the photon flux^[6]^ (see the Experimental Section in Supporting Information). The highest yield is achieved under 410 nm LED light irradiation. The most significant LSPR absorption of Ag NPs in this photocatalyst appears at 405 nm and matches well with the highest yield of the reaction. The dependence of AQY on the irradiation wavelengths generally follows the light absorption of the catalyst. It evidences that the enhancement of the catalytic performance is mainly due to the LSPR absorption of Ag NPs. The intuitive inference is that greater light absorption produces more hot electrons when hot electrons generated by the LSPR absorption drive the reaction. Besides, the more intense LSPR light absorption results in higher intensity of the EM near‐field around the NPs, which leads to more adsorbed *β*‐O‐4 molecules, and a higher rate of hot‐electron excitation. Hence, the AQY effectively depends on the light absorption of the catalyst at a low light intensity (0.2 W cm^−2^). But a deviation that falls below the absorption at a longer wavelength under a low reactant concentration (5×10^−3^ M) and high light intensity (0.5 W cm^−2^) can be observed (the AQY over Ag_H_‐S‐Al_2_O_3_ catalyst under 410 nm can reach about 32 times of that under 650 nm, as displayed in Figure 3c). The phenomenon suggests that LSPR‐induced adsorption plays an important role in promoting the catalysis when the reactant concentration is low, and light intensity is high. This is consistent with the kinetic results displayed in Figure 3d.

We evaluated the kinetic parameters of 1‐phenoxy‐2‐phenylethane conversion processes to demonstrate the light‐induced reaction mechanism. Figure 3d shows that the experimental data fit Michaelis–Menten type curves.^[25]^ The Michaelis constant *K*_M_ values are summarised in the figure. The *K*_M_ values are equal to 1‐phenoxy‐2‐phenylethane concentration, at which the reaction rate of the β‐O‐4 compound conversion is at half‐maximum. A smaller *K*_M_ value indicates a higher affinity of the catalytic sites toward the reactant molecules.^[25]^ The derived *K*_M_ values for Ag_H_‐S‐Al_2_O_3_ under light irradiation were much lower than that of Ag_L_‐S‐Al_2_O_3_, suggesting the former had a higher affinity toward the *β*‐O‐4 compound than the latter. The affinity enhancement can be attributed to the light‐promoted chemisorption in the Ag_H_‐S‐Al_2_O_3_ catalyst.

Moreover, for the Ag_H_‐S‐Al_2_O_3_ catalyst, the *K*_M_ value under 440 nm wavelength light was 0.017 mM, approximately 1/3 of that under 650 nm light, indicating a significantly lower *β*‐O‐4 compound concentration was required under the LSPR absorption of Ag NPs for achieving a maximum activity. It is reasonable that more intensive EM fields can be generated under a 440 nm wavelength light, resulting in the higher chemisorption of 1‐phenoxy‐2‐phenylethane and reaction rate. To further investigate the influence of the interaction between the reactant and catalyst on the conversion, we studied the time course of the reactant using a Ag_H_‐S‐Al_2_O_3_ catalyst under irradiation with two different wavelengths (440 nm and 650 nm) at the same intensity (0.5 W cm^−2^). As seen in Figure S7, under the 440 nm light irradiation, the conversion increased dramatically as the reaction proceeded, reaching a maximum of 99 % at 20 h. The conversion achieved under the 650 nm light was lower than 40 % after 20 h of reaction, where further increase in reaction time resulted in slow or little change in the reactant conversion. As a result, the conversion obtained under 650 nm light for 100 h (88 %) was still lower than that under 440 nm light for 20 h. The study corroborates that we can tune the affinity of the *β*‐O‐4 compound to the catalysts with the irradiation wavelength and the number‐density of Ag NPs on the catalyst, which significantly influence the photocatalytic efficiency.

The hot electrons generated by light absorption also play an essential role in catalysing the cleavage. As shown in Figure 2c, the reaction is suppressed totally in an air atmosphere (1^st^ column). It is known that the work function of Ag noticeably increases when it adsorbs O_2_, and LSPR‐induced energetic electrons on the metal NPs surface can transfer to the unoccupied orbital of adsorbed O_2_.[^1b^, ^26^] This process likely hinders *β*‐O‐4 compounds from gaining hot electrons.

**Figure 4:**
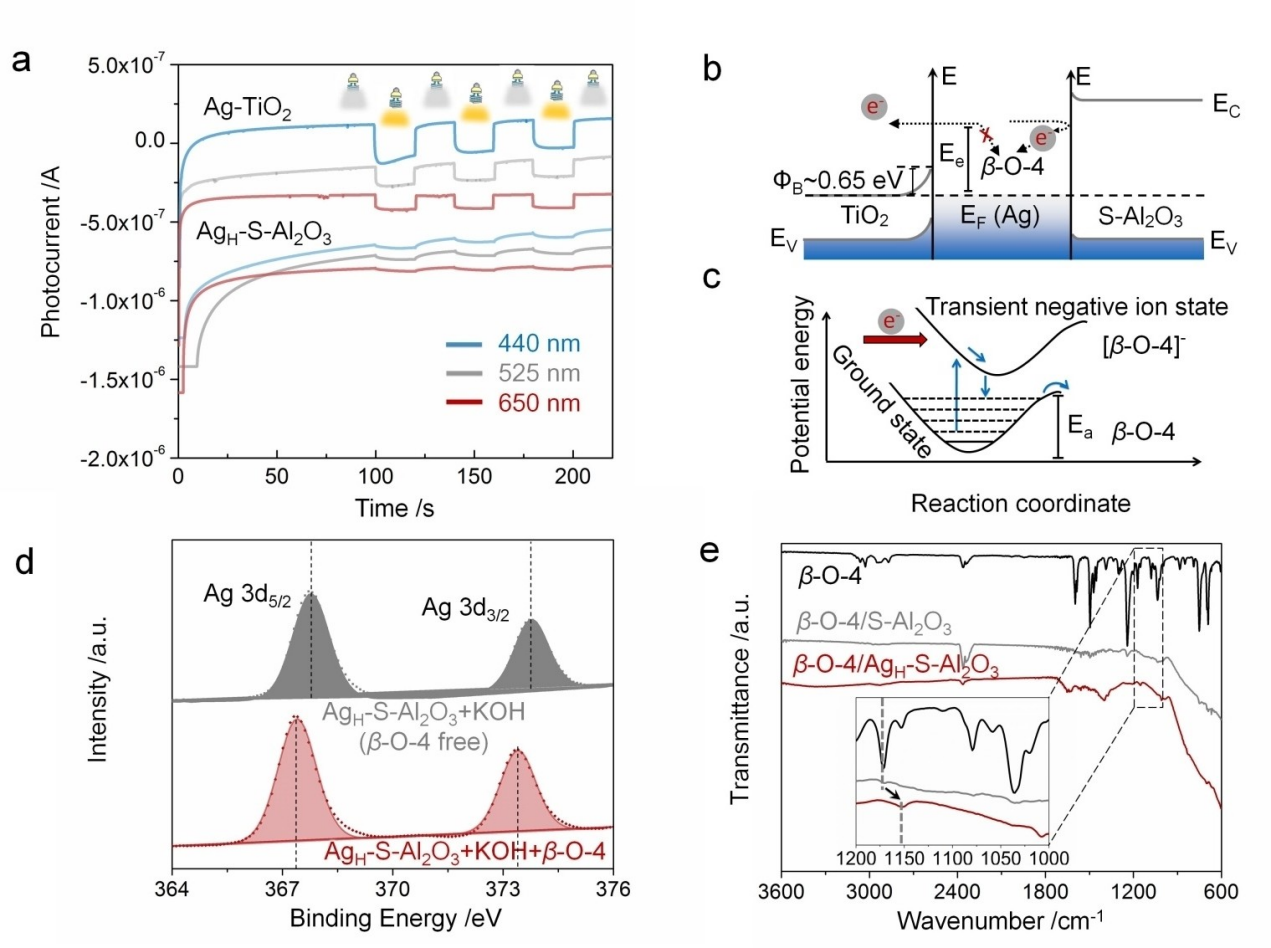
a) Photo‐switching characteristics of Ag‐TiO_2_ and Ag_H_‐S‐Al_2_O_3_ catalysts under varying light wavelengths. The data were collected with a three‐electrode system (counter electrode: Pt; reference electrode: Calomel; electrolyte: 0.1 M NaSO_4_) under three different wavelengths LED as the light sources; the light intensity was 0.1 W cm^−2^. b) The schematic drawing of the route alteration of hot electron transfer from plasmonic Ag NPs due to the support. The barrier height of the Ag‐TiO_2_ catalyst was calculated via the photoresponse measurements, where the raw data is given in Figure S8. c) Schematic illustrating the change of the potential energy surface caused by a hot electron transfer from a metal NP to an adsorbed molecule. *E*_a_ represents activation energy. d) XPS of the Ag_H_‐S‐Al_2_O_3_ photocatalyst after 1 h of light reaction in the system with (red) and without (grey) *β*‐O‐4 compound. e) FTIR spectra of *β*‐O‐4 compound (black), *β*‐O‐4 compound adsorbed on S‐Al_2_O_3_ support (grey) and Ag_H_‐S‐Al_2_O_3_ catalyst (red).

Further evidence that the reaction involves hot electrons is that when loaded on anatase TiO_2_, a typical photocatalytic semiconductor, Ag NPs give lower conversions (2^nd^ column, Figure 2c). The energy barrier at noble metal/anatase TiO_2_ junctions is much lower than that at the metal/Al_2_O_3_ junctions.^[27]^ We measured the photocurrent response (Figure 4a and Figure S8). The Schottky barrier height of Ag‐TiO_2_ was about 0.65 eV, calculated by the linear fitting of the normalised photocurrent to the photon energy.^[28]^ In contrast to Ag‐TiO_2_, the Ag_H_‐S‐Al_2_O_3_ catalyst produced much lower photocurrents independent of light wavelength (Figure 4a). These results imply that S‐Al_2_O_3_ support provides a high barrier to confine excited electrons on the surface of Ag NPs, which are readily available for transfer to the reactant. It appears that the possibility of injecting light‐induced hot electrons from the NPs into TiO_2_ reduces the availability of the hot electrons for driving H abstraction and the *β*‐O‐4 cleavage on the NPs surface (Figure 4b).

These results indicate that the hot electrons can inject into the reactant molecules adsorbed on the NPs. It is accepted that the light‐metal interaction creates hot electrons, which can be transferred to unpopulated orbitals of molecules adsorbed on the metal surface, forming TNI species.^[29]^ The TNI‐metal system is on a higher potential energy surface than that of the adsorbed molecules on a metal surface in the dark (Figure 4c)[^29a^, ^29b^] and can result in the chemical bond‐breaking within the adsorbed molecule or desorption of the adsorbate.

**Figure 5:**
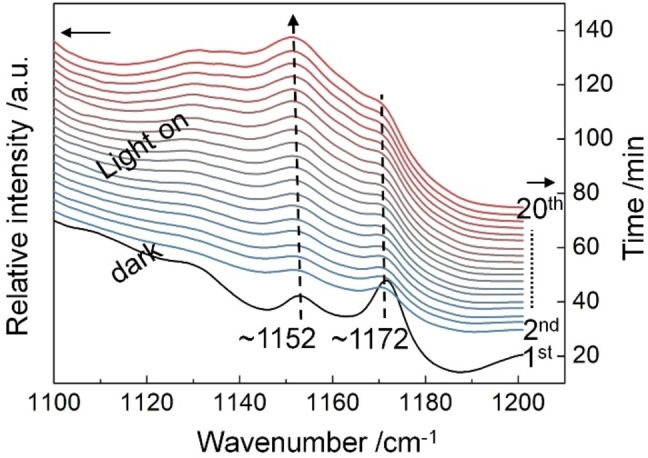
DRIFT spectra of the mixture of *β*‐O‐4 model compound and Ag_H_‐S‐Al_2_O_3_ catalyst irradiated under a light (405 nm wavelength at 0.5 W cm^−2^ of intensity) for various times in argon atmosphere at 363 K. The first spectrum (the black line) was recorded after stabilising the setup in the dark for 20 min, then the light was turned on, and the second spectrum was recorded at 10 min. The remained data were collected every 2.5 min under irradiation.

X‐ray photoelectron spectroscopy (XPS) and FTIR results confirm a strong interaction between Ag NPs and 1‐phenoxy‐2‐phenylethane. The strong adsorption of the *β*‐O‐4 compound on the Ag NP surface causes the negative shift of the binding energy of Ag (Figure 4d). Figure 4e shows the peak at 1172 cm^−1^ (characteristic C−O bond stretch mode of the ether) shifted to 1152 cm^−1^ in the 1‐phenoxy‐2‐phenylethane/Ag_H_‐S‐Al_2_O_3_ sample. The in situ diffuse reflectance infrared transform spectroscopy (DRIFT) analysis of the mixture of the reactant and catalyst was carried out to investigate the shift. As displayed in Figure 5, the peaks located at about 1152 and 1172 cm^−1^ are the characteristic C−O bond stretch mode of the ether. They can be ascribed to the chemisorption and physisorption of the *β*‐O‐4 model compound on the Ag_H_‐S‐Al_2_O_3_ catalyst, respectively. The peaks at around 1152 cm^−1^ correspond to *v*(C−O) stretching of the reactant via chemisorption and are slightly redshifted due to the changing from a more end‐on to a more side‐on adsorption orientation.^[30]^

Strikingly, the intensity ratio of the peaks at 1152 and 1172 cm^−1^, I_1152_/I_1172_, increases obviously under irradiation (Figure 5 and Figure S9a), indicating the light‐induced chemisorption for the reactant on Ag_H_‐S‐Al_2_O_3_ catalyst. The detailed spectroscopy analysis under in situ irradiation is consistent with our previous discussion on the spectra in Figure 4e. The chemisorption of the reactant on the catalyst is an important step for heterogeneous catalysis. It results in a significant increase in the reactant concentration around the active sites (Ag NPs in the present study), reduces the activation energy barrier of the reaction, and accelerates the transfer of excited electrons and absorbed energy from the Ag NPs to the reactant. The light‐enhanced physisorption^[12]^ and chemisorption^[7]^ are unique features of plasmonic catalysis reported recently. With the strong adsorption of the *β*‐O‐4 compound on the Ag NPs, the transfer of hot electrons of the illuminated Ag NPs to the adsorbed *β*‐O‐4 model molecule is feasible.

**Figure 6:**
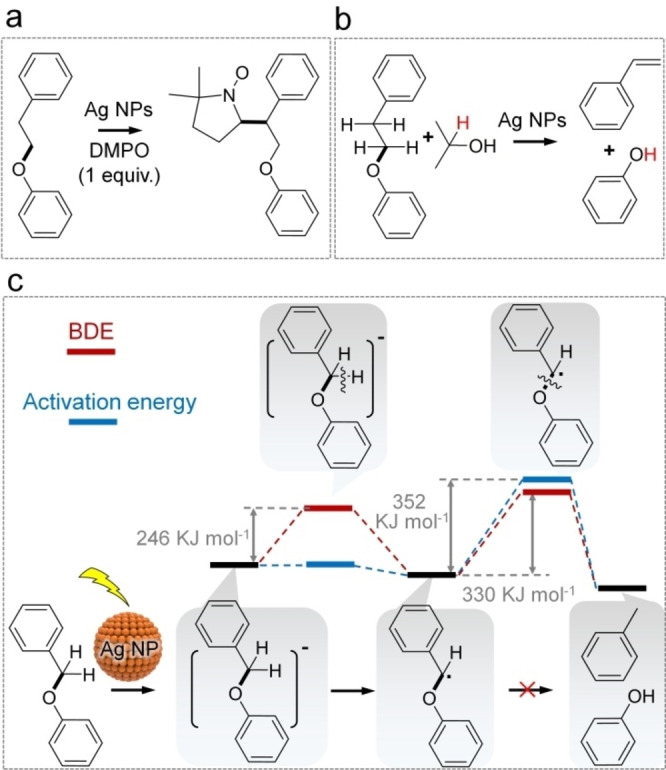
a) The proposed radical process is supported by adding DMPO to the standard reaction system with EPR and LC‐MS detection (Figures S8a and S9). b) Isopropanol functions as a hydrogen‐donating solvent in the reaction. c) The calculated activation energy of TNI, dissociation energy of C_α_−H bonds and alkyl ether C−O bonds in the compound with *α*‐O‐4 linkage after hot electrons are transferred from illuminated Ag NPs.

The H‐abstraction shown in Figure 1 should yield carbon‐centred radicals. Figure 2c shows that the conversion of 1‐phenoxy‐2‐phenylethane is inhibited to different degrees when radical scavengers, 4‐hydroxy‐2,2,6,6‐tetrame‐thylpiperidine 1‐oxyl (TEMPO) and 5,5‐dimethyl‐1‐pyrroline‐*N*‐oxide (DMPO), were added to the reaction, respectively. We surmised that a radical‐mediated catalytic pathway is, therefore, likely involved (Figure 6a). DMPO reduces the conversion of *β*‐O‐4 compound more significantly than 4‐Hydroxy‐TEMPO (4^th^ vs 3^rd^ columns, Figure 2c). The electron affinity of DMPO is much lower than that of TEMPO,^[29c]^ making DMPO more suited to capture radicals in reductive conditions (TEMPO could be consumed much faster by hydrogen atoms from alcohol). The results of electron paramagnetic resonance (EPR) spectra and liquid chromatography‐mass spectrometry (LC‐MS) suggest that benzylic radical (C_14_H_13_O) was captured by DMPO (Figure S10a and Figure S11), confirming the formation of carbon‐centred radicals produced by the C_α_−H or C_β_−H bond cleavage in the photocatalytic system.

The simulation results in Figure 6c also indicate that although a similar BDE (246 kJ mol^−1^) is needed for C_α_−H bond cleavage in benzyl phenyl ether with an *α*‐O‐4 linkage under a transient anion state, H‐abstraction increases the BDE for the cleavage *α*‐O‐4 linkage (from 263 increased to 330 kJ mol^−1^, Table S1 and Figure 6c). Besides, the activation energy barrier for converting the C_α_ radical of the linkage is as high as 352 kJ mol^−1^. These data explain the negligible conversion of *α*‐O‐4 compound over the Ag_H_‐S‐Al_2_O_3_ catalyst (Figure 2d). The H‐abstraction from the benzylic C_*α*_−H bond over the illuminated Ag NPs provides a unique parameter to control C_β_−O bond cleavage for the selectivity to the less hydrogenated products.

We also conducted the photocatalysis using IPA‐d8 as the solvent. The NMR analysis of the product (Figure S12) shows that no signals ascribed to OH groups of phenol can be found, suggesting the formation of the phenol‐OD compound in the reaction. Hence, isopropanol is an H‐donor for the hydrogenolysis of 1‐phenoxy‐2‐phenylethane. The donated hydrogen prevents oxidation of the phenolic product, as Figure 6b illustrates.

Methanol and tetrahydrofuran have a weaker ability to donate hydrogen compared to isopropanol. When they were the solvents, benzoquinone was produced instead under mild conditions (Figures S5 and S6).

**Scheme 1:**
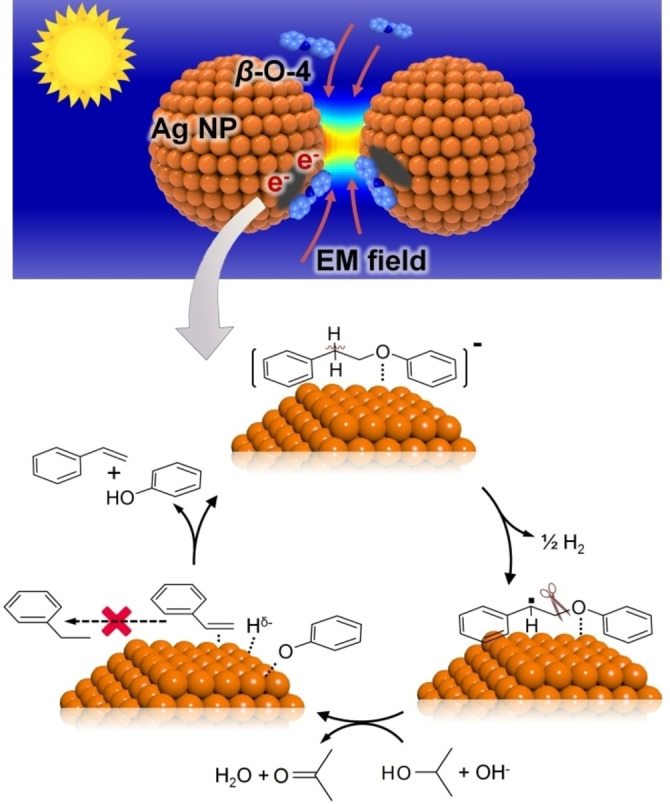
Proposed mechanism of C−O bond cleavage of *β*‐O‐4 lignin model compound by Ag_H_‐S‐Al_2_O_3_ catalyst.

Based on the discussions above, we propose a reaction mechanism for the photocatalytic cleavage of the alkyl‐aryl ether C−O bonds by the supported Ag NPs. As shown in Scheme 1, under visible‐light irradiation, the closely packed Ag NPs can generate a strong EM near‐field, promoting the chemisorption of the *β*‐O‐4 compound molecules onto the catalyst with a side‐on configuration. The enhanced chemisorption contributes to the superior photocatalytic activity of the Ag_H_‐S‐Al_2_O_3_ catalyst kinetically. The intense LSPR absorption of Ag NPs generates hot electrons that transfer to the adsorbed *β*‐O‐4 model molecules. The *sp* ^3^ hybridised benzylic C_α_−H bond in the resultant transient anionic species is weakened.

Ag NPs can abstract the H atom from the C_α_−H bond under moderate conditions, yielding a carbon‐centred radical. Compared with the energy needed to cleave the C_β_−O bond directly (≈289 kJ mol^−1^),^[18b]^ the energy required to cleave the C_β_−O bond of the carbon‐centred radical is significantly lower (≈46 kJ mol^−1^). Homolytic cleavage of the radical yields an adsorbed phenoxy and styrene. The adsorbed phenoxy combines with the hydrogen species donated from IPA, yielding phenol. In the cleavage of the *β*‐O‐4 compounds using the Ag_H_‐S‐Al_2_O_3_ catalyst, styrene was obtained instead of ethylbenzene. It has been reported that the generation of H^δ−^ species from hydrogen sources such as H_2_ is critical for achieving high selectivity toward styrene.^[31]^ In other words, the polar hydrogen species (H^δ−^) is not active for C=C bond reduction. Polar H^δ−^ species were produced on illuminated Ag NPs via heterolytic dissociation of hydrogen sources.[^31^, ^32^] When the light‐generated hot electrons transfer from the NPs to the adsorbed molecules, the Ag NPs with electron deficit favour hosting the H^δ−^ species. This explains why the styrene was not hydrogenated on the illuminated Ag NPs. We also conducted the experiment of styrene hydrogenation using a series of catalysts. As shown in Table S5, hydrogenation of styrene cannot be achieved by Ag_H_‐S‐Al_2_O_3_ and S‐Al_2_O_3_‐Ni^2+^ (monometallic catalysts, Entries 1–2 and 4–5). Light irradiation on S‐Al_2_O_3_‐Ni^2+^ cannot generate hot electrons or H species from hydrogen sources. In comparison, obvious conversion of styrene to ethylbenzene is observed using Ag_H_‐S‐Al_2_O_3_‐Ni^2+^ catalyst even at a shorter reaction time (Entries 3 and 6 in Table S5). We have reported that a Ni^2+^/Ni^0^ transformation can occur in the Ag_H_‐S‐Al_2_O_3_‐Ni^2+^ catalyst under reductive reaction conditions.^[7]^ The Ni species in a metallic state can yield nonpolar H species that are active for C=C bond reduction.^[31]^ Hence, it is rational that supported Ag NPs have a poor hydrogenation ability that cannot convert styrene into ethylbenzene using IPA or H_2_ as the hydrogen sources.

On the other hand, the illuminated plasmonic metal NPs can abstract the hydrogen from C_α_−H of *α*‐O‐4 linkage (Figures S10b and S13). The calculated BDE of the C_α_−O bond of the resultant carbon‐centred radical is 330 kJ mol^−1^. Thus, plasmon‐driven benzylic C_α_−H bond activation cannot promote the cleavage of the *α*‐O‐4 model molecule. We have reported photocatalysts of strong reduction power,^[7]^ Ag_H_‐S‐Al_2_O_3_‐Ni^2+^ and Au_H_‐S‐Al_2_O_3_‐Ni^2+^, prepared by introducing Ni^2+^ ions. The hot electron generated on the illuminated plasmonic metal NPs migrated to Ni^2+^ sites of the catalysts, and the reduced Ni^0^ sites can convert *α*‐O‐4 and *β*‐O‐4 model molecules completely, yielding phenol, toluene and ethylbenzene (Table S6).

The Ag_H_‐S‐Al_2_O_3_ and Au_H_‐S‐Al_2_O_3_ photocatalysts exhibited a moderate reduction power and a new reaction channel that selected for cleavage of the *β*‐O‐4 linkage. Focusing on the model reaction of 1‐phenoxy‐2‐phenylethane, this demonstrated how practical the effect can be. The plasmon‐driven mechanism was harnessed to directly yield an unsaturated product such as styrene, which is a far more valuable chemical commodity than the saturated small‐molecule products that are ordinarily produced using similar catalytic methods.

## Conclusion

The present study reveals a novel property of plasmonic catalysts such that illuminated Ag NPs can abstract the hydrogen at C_α_−H of the alkyl‐aryl ether molecules with *β*‐O‐4 by transferring hot electrons to the adsorbed ether molecules. The intense near EM fields of the Ag NPs promote the ether chemisorption on the catalyst and the electron transfer. The bond dissociation energy of the C(sp^3^)−H bond of the C_α_ atom is much lower than that of other C atoms in the transient negative ion and that in the ether molecule. The H‐abstraction weakens the C_β_−O bond significantly, facilitating a homolytic cleavage under mild conditions. Polar H^δ−^ species were extracted from alcohols on illuminated Ag NPs. The relatively modest hydrogenation ability of the species enables the reductive cleavage of alkyl‐aryl ether bonds to selectively generate unsaturated products, such as styrene, which thermal catalytic reaction processes can hardly yield. The plasmon‐driven selective activation of the *sp* ^3^ hybridised benzylic C_α_−H bond also provides an efficient strategy for selective bond cleavage in synthesis under mild conditions and visible‐light irradiation. Applying the Ag_H_‐S‐Al_2_O_3_ photocatalyst for the depolymerisation of lignin is ongoing. The challenge is effective contact between the plasmonic NPs and *β*‐O‐4 linkage due to the poor solubility of real lignin in IPA.

## Conflict of interest

The authors declare no conflict of interest.

1

## Supporting information

As a service to our authors and readers, this journal provides supporting information supplied by the authors. Such materials are peer reviewed and may be re‐organized for online delivery, but are not copy‐edited or typeset. Technical support issues arising from supporting information (other than missing files) should be addressed to the authors.

Supporting Information

## Data Availability

The data that support the findings of this study are available in the supplementary material of this article.
