# Supplementary material for: Plasmonic Silver‐Nanoparticle‐Catalysed Hydrogen Abstraction from the C(sp 3 )−H Bond of the Benzylic Cα atom for Cleavage of Alkyl Aryl Ether Bonds
Source: Angew Chem Int Ed Engl. 2022 Dec 16;62(4):e202215201. doi: 10.1002/anie.202215201 (PMC10108273; doi:10.1002/anie.202215201)
Supplement: Supplementary file 1 — Supporting Information [file ANIE-62-0-s001.pdf]

Angewandte Chemie
Eine Zeitschrift der Gesellschaft Deutscher Chemiker

Supporting Information

# Plasmonic Silver-Nanoparticle-Catalysed Hydrogen Abstraction from the C($sp^3$)–H Bond of the Benzylic $C_\alpha$ atom for Cleavage of Alkyl Aryl Ether Bonds

*P. Han, X. Mao, Y. Jin, S. Sarina, J. Jia, E. R. Waclawik*, A. Du, S. E. Bottle, J.-C. Zhao, H.-Y. Zhu**

**Table of Contents**

Experimental Procedures ..... 3
Results and Discussion ..... 6
Table S1. ..... **Error! Bookmark not defined.**
Table S2. ..... 7**!Unexpected End of Formula**
Table S3. ..... 8
Table S4.. ..... 9
Table S5.. ..... 10
Table S6.. ..... 11
Figure S1.. ..... 12
Figure S2. ..... 13
Figure S3. ..... 14
Figure S4.. ..... 15
Figure S5.. ..... 16
Figure S6.. ..... 17
Figure S7.. ..... 18
Figure S8.. ..... 19
Figure S9.. ..... 20
Figure S10.. ..... 21
Figure S11.. ..... 22
Figure S12.. ..... 23
Figure S13.. ..... 24
Figure S14.. ..... 25
Figure S15.. ..... 26
Figure S16.. ..... 27
Author Contributions ..... 28
References ..... 29

## Experimental Procedures

### *Materials.*

The chemicals were purchased from commercial suppliers and used as provided: benzyl phenyl ether (Aladdin, >98%), 1-phenoxy-2-phenylethane (Aladdin, 98%), 2-phenoxyacetophenone (Aladdin, >98%), 2-phenoxy-1-phenylethanol (Aladdin, 98%), 1-methoxy-4-(2-phenoxyethyl)benzene (Foshan Haer Biotechnology Co., Ltd, >95%), 1-methyl-4-(2-phenylethoxy)benzene (Foshan Haer Biotechnology Co., Ltd, >95%), 4-hydroxy-2,2,6,6-tetramethylpiperidine 1-oxyl (Adamas, >98%), 5,5-dimethyl-1-pyrroline-*N*-oxide (Adamas, >98%), styrene (Adamas, 99%), phenol (Adamas, 99%), sodium sulfate anhydrous (Keshi, >99%), sodium silicate nonahydrate (Hushi SCR, AR), titanium dioxide, anatase (Aladdin, 99%, <100 nm), potassium hydroxide (Sigma-Aldrich, >99.99%), silver(I) nitrate (Hushi SCR, >99.8%), hydrogen tetrachloroaurate(III) trihydrate (Adamas, >99.9%), nickel(II) nitrate hexahydrate (Scharlau, >98%), sodium borohydride (Sigma-Aldrich, >98%), sodium aluminate (Sigma-Aldrich, anhydrous), isopropanol (TCI, >99.5%), isopropanol-d8 (Acros, >99%), dimethyl sulfoxide-d6 (Adamas, 99.8%), 2-butanol (Greagent, >99.9%), ethanol (Hushi SCR, >99.7%), capryl alcohol (Adamas, 99%), acetone (Hushi SCR, >99.5%), methanol (Hushi SCR, >99.5%), tetrahydrofuran (Macklin, 99%), toluene (Fisher, >99.99%, GC assay), N,N-dimethyl formamide (Sigma-Aldrich, >99.8%, anhydrous), α,α,α-trifluorotoluene (Sigma-Aldrich, >99%, anhydrous), nitric acid (Ajax Finechem, 68-70%), PEO surfactant ($C_{12-14}H_{25-29}O(CH_2CH_2O)_5H$) (Sigma-Aldrich), (3-aminopropyl)trimethoxysilane (Sigma-Aldrich, >97%), Ar (Supagas, >99.99%), $H_2$ (Supagas, >99.99%).

### *Catalyst Preparation.*

*γ*-$Al_2O_3$ nanofibres were used as catalyst support and prepared by the previously published method.[1] Boehmite (AlOOH) nanofibers were prepared from $NaAlO_2$ and converted to *γ*-$Al_2O_3$ fibres by calcination at 450°C for 5 h. (3-aminopropyl)trimethoxysilane was then grafted on the *γ*-$Al_2O_3$ fibres in refluxing toluene for 40 h. The grafted samples were collected by washing with $H_2O$ and ethanol and then drying at 60°C in a vacuum ($Al_2O_3$-silane-$NH_2$ labelled S-$Al_2O_3$).

Ag NPs were prepared on different supports via the same impregnation-reduction method. For example, the synthesis of 4.3 wt% of Ag NPs on S-$Al_2O_3$ support is the following: 1.0 g of S-$Al_2O_3$ was dispersed into 133 mL of DI water under sonication before vigorously stirring for 20 min. 92.6 mL of $AgNO_3$ aqueous solution (0.01 M) was added to the suspension instantly and stirred for a further 20 min. Then 246 mL of $NaBH_4$ (ten times the molar amount of added metal) aqueous solution (0.038 M) was added at a speed of 2.5 mL·min$^{-1}$ to the suspension under stirring. The suspension was aged for 24 h, and the solid was then filtrated, washed with water and ethanol, and dried at 60°C in a vacuum. The obtained sample was labelled $Ag_H$-S-$Al_2O_3$.

The silica-coated $Ag_H$-S-$Al_2O_3$ catalyst ($SiO_2$@$Ag_H$-S-$Al_2O_3$) was prepared according to the method reported.[2] Two solutions were freshly prepared before the coating experiment. Solution A: 18.3 μL of

(3-Aminopropyl)trimethoxysilane (APTMS, Adamas, 98%) was added to a 100-mL volumetric flask and filled with Milli-Q water to the mark. Solution B: 1.257 g of sodium silicate nonahydrate was added to a beaker and filled with ~20 mL of Milli-Q water. After the dissolution, 60 mL of hydrochloric acid solution (0.01 M) was added to the beaker, followed by adjusting the pH to ~10.2 using hydrochloric acid solution (~1 M). The solution was then transferred to a 100-mL volumetric flask and filled with Milli-Q water to the mark. Preparation of $SiO_2$@$Ag_H$-S-$Al_2O_3$: about 30 mg of $Ag_H$-S-$Al_2O_3$ catalyst was placed in a beaker and dispersed in 30 mL of Milli-Q water by sonication for 2 minutes. Then 2 mL of solution A was added and stirred for 15 min, followed by adding 16 mL of solution B and stirred further for 5 min. The beaker was then heated at 90°C for 60 min in an oil bath. Finally, the mixture was separated by centrifugation (4500 rpm for 5 min), washed with water and ethanol, and dried at 60°C in a vacuum oven overnight to obtain the silica-coated sample.

***Photocatalytic Reactions.***

A light reaction chamber was used for the photocatalytic reaction. A standard reaction procedure used a 10 mL glass tube (Pyrex) as the reaction container. The tube was filled with argon and sealed with a rubber septum cap before adding the reactants and catalyst. Then the tube was placed in an oil bath on a magnetic stirrer with stirring and illuminated under a halogen lamp (Philips Industries: 500W, wavelength in the range 400-750 nm) or LED light. The reaction temperature in the dark and under irradiation was the same. The tube was wrapped with aluminium foil to avoid light exposure in a typical dark reaction. After the reaction, the mixture was filtered through a Millipore filter (0.22 µm of pore size) to remove the solid photocatalyst. The filtrate was analysed by an Agilent 6890 gas chromatography (GC) equipped with an HP-5 column. The acetone was determined and calibrated by a Shimadzu GC2030 equipped with an SH-Rxi-5sil MS column. An Agilent HP5973 mass spectrometer was used to identify the product. The EIS spectra of the radical adduct were obtained from a Shimadzu LC-MS2020.

$$Aapparent\ quantum\ yields\ (AQY) = \frac{Conv_{light} - Conv_{dark}}{n} \times 100\%$$

where the $Conv_{\text{light}}$ and $Conv_{\text{dark}}$ are the conversion of reactant under light irradiation and dark conditions, respectively; $n$ is the number of incident photons.

***Catalyst Characterisation.***

Metal contents in catalysts were obtained by inductively coupled plasma mass spectrometry (ICP-MS) on an Agilent 8900. Before analysis, the powder samples were dissolved in $HNO_3$ (70%) and diluted with deionised water. FT-IR measurements were conducted on Perkin-Elmer Spectrum. The samples were prepared in KBr pellets and stabilised under controlled relative humidity before acquiring the spectrum. The particle size and morphology of the catalyst samples were characterised with a

JEOL2100 transmission electron microscope equipped with a Gatan Orius SC1000 CCD camera. A Shimadzu UV-2600 was used to collect the diffuse reflectance UV-visible (DR-UV-vis) spectra of the samples. X-ray photoelectron spectroscopy (XPS) measurements were carried out using the AXIS SUPRA, Kratos Analytical. The current-time curves were recorded on a CHI660E electrochemical workstation. EPR spectra were recorded with a JES-FA 200 spectrometer operating in the X-band mode. The diffuse reflectance infrared transform spectroscopy (DRIFT) was measured on a Nicolet iS50 spectrometer, which incorporates a single-bounce diamond ATR module with a dedicated MCT detector (Thermo Fisher Scientific Inc., Madison, WI, USA).

***Computational Details.***

The near-field enhancement of dimer Ag NPs was simulated using the finite difference time domain (FDTD) method for solving Maxwell's equations. The FDTD solutions from Lumerical, Inc. were used as the package. The refractive index of Ag NPs was taken from the CRC database provided by the package.

Self-consistent periodic DFT calculations were performed using Vienna ab initio Simulation Package (VASP).[3] The interaction between ionic cores and electrons was described by the projector-augmented wave (PAW) method,[4] and the exchange-correlation energy was calculated within the generalised gradient approximation (GGA) and Perdew-Burke-Ernzerhof (PBE) functional.[5] The Kohn-Sham equations were solved using a plane-wave basis set with a kinetic energy cutoff of 400 eV. The convergence criteria for self-consistent electronic iteration were set to $1.0 \times 10^{-4}$ eV, and the atomic positions were optimised using a conjugate gradient algorithm until atomic forces were smaller than 0.03 eV. The properties of isolated chemical compounds were calculated using a 25 × 25 × 25 Å cubic unit cell. A 1 × 1 × 1 Monkhorst-Pack *k*-point grid was used to sample the surface Brillouin zone accordingly.

**Results and Discussion**

**Table S1.** Calculated dissociation energy of typical bonds in the *β*-O-4 model compound by a density functional theory method.

| Entry | Target molecules | Target bond for cleavage | BDE /KJ·mol$^{-1}$ |
|---|---|---|---|
| 1 | 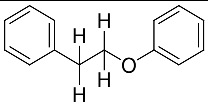 | $C_\alpha$-H | 470.4 |
| 2 | 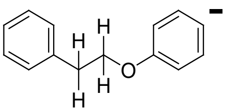 | $C_\alpha$-H | 250.47 |
| 3 | 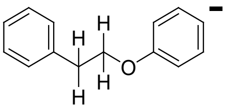 | $C_\beta$-H | 329.01 |
| 4 | 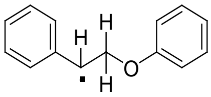 | $C_\beta$-O | 46.17 |
| 5 | 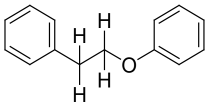 | $C_\beta$-O | 289[6] |
| 6 | 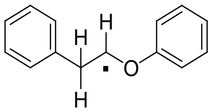 | $C_\beta$-O | 371.10 |
| 7 | 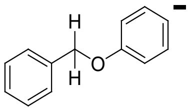 | $C_\alpha$-H | 246.26 |
| 8 | 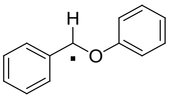 | $C_\alpha$-O | 329.64 |
| 9 | 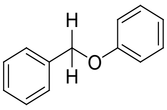 | $C_\alpha$-O | 263.03 |

The target molecule in Entries 2, 3 and 7 are the transient state of lignin model compounds formed after accepting one electron from the illuminated Ag NPs.

**Table S2.** ICP-MS analysis of metal content in the photocatalysts and their catalytic performances for *β*-O-4 lignin model compound cleavage.

| Entry | Samples | Ag/Au content wt% | Conversion /% |
|---|---|---|---|
| 1 | $Ag_L$-S-$Al_2O_3$ | 1.3 | 10 |
| 2 | $Ag_H$-S-$Al_2O_3$ | 4.3 | 96 |
| 3 | Ag-$Al_2O_3$ | 7.2 | 95 |
| 4 | Ag-$ZrO_2$ | 7.2 | 95 |
| 5 | Ag-$TiO_2$ | 7.3 | 19 |
| 6 | Ag-$CeO_2$ | 6.5 | 18 |
| 7 | $Au_L$-S-$Al_2O_3$ | 0.7 | *25 |
| 8 | $Au_H$-S-$Al_2O_3$ | 2.5 | 58 |
| 9 | S-$Al_2O_3$ | 0 | 0 |
| 10 | $Al_2O_3$ | 0 | 0 |
| 11 | $ZrO_2$ | 0 | 0 |
| 12 | $TiO_2$ | 0 | 0 |
| 13 | $CeO_2$ | 0 | 0 |

10 mg of each powder sample was dissolved in 1 mL of $HNO_3$ (70 wt%) and then diluted with 9 mL of deionised water. Before the analysis, the mixture was filtered through a 0.22 μm filter to obtain the solution. Reaction conditions: 1-phenoxy-2-phenylethane (*β*-O-4 model, 0.05 mmol), KOH (0.15 mmol), 2 mL of isopropanol solvent, 1 atm Ar atmosphere, 10 mg of catalysts, under irradiation of 0.5 W $cm^{-2}$ from a LED lamp of 440 nm wavelength for 20 h at 90±2°C.*35 mg of catalyst was used.

**Table S3.** Influence of solvents on the catalytic performance of $Ag_H$-S-$Al_2O_3$ catalyst for the cleavage of 1-phenoxy-2-phenylethane.

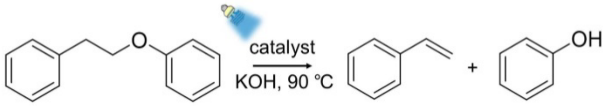


| Entry | The second solvent | Relative polarity [a] | Condition | Conversion (%) |
|---|---|---|---|---|
| 1 | Methanol | 0.762 | Light | 88 |
| 2 | Capryl alcohol | 0.537 | Light | 34 |
| 3 | 2-butanol | 0.506 | Light | >99 |

Reaction conditions: 1-phenoxy-2-phenylethane (*β*-O-4 model, 0.05 mmol), KOH (0.15 mmol), 2 mL of IPA (relative polarity: 0.546) and the second solvent (1:1 v/v), 1 atm argon atmosphere, 10 mg of catalysts, under irradiation of 0.5 W $cm^{-2}$ from a LED lamp of 440 nm wavelength for 20 h at 90±2°C. [a]The relative polarity data can be found in the reference.[7]

**Table S4.** The effect of S-$Al_2O_3$ added amount on the adsorption of the *β*-O-4 compound.

| Entry | Added amount of S-$Al_2O_3$ /mg | *β*-O-4 compound area percentage /% | Reactant concentration change /% |
|---|---|---|---|
| 1 | 0 | 0.142 | 0 |
| 2 | 10 | 0.142 | 0 |
| 3 | 20 | 0.142 | 0 |
| 4 | 30 | 0.142 | 0 |

In a typical procedure, S-$Al_2O_3$ sample was added to 2 mL of *β*-O-4/2-butanol ($5 \times 10^{-3}$ M) solution, filled with argon and sealed. The adsorption was conducted at 90°C for 8 h, the same temperatures designated for the reaction. A 440 nm LED light at 0.5 $W \cdot cm^{-2}$ was used as the light source. Only the *β*-O-4 compound and 2-butanol can be detected by GC, for KOH is absent in the experiment.

**Table S5.** Catalytic performances of the photocatalysts for the hydrogenation of styrene.

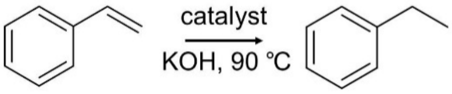


| Entry | Catalysts | Reaction time (h) | Condition | Conversion (%) |
|---|---|---|---|---|
| 1 | $Ag_H$-S-$Al_2O_3$ | 2 | Light | 0 |
| | | | Dark | 0 |
| 2 | S-$Al_2O_3$-$Ni^{2+}$ | 2 | Light | 0 |
| | | | Dark | 0 |
| 3 | $Ag_H$-S-$Al_2O_3$-$Ni^{2+}$ | 1 | Light | >99 |
| | | | Dark | 4 |
| *4 | $Ag_H$-S-$Al_2O_3$ | 2 | Light | 0 |
| | | | Dark | 0 |
| *5 | S-$Al_2O_3$-$Ni^{2+}$ | 2 | Light | 0 |
| | | | Dark | 0 |
| *6 | $Ag_H$-S-$Al_2O_3$-$Ni^{2+}$ | 1 | Light | 10 |
| | | | Dark | 16 |

Reaction conditions: styrene (0.05 mmol), KOH (0.15 mmol), 2 mL of IPA solvent, 1 atm argon atmosphere, 20 mg of catalysts, 1.1 W $cm^{-2}$ of light intensity (400-750 nm wavelength), at 90°C. *using toluene instead of IPA as the solvent, 1 atm $H_2$ atmosphere. The conversion was measured by GC analysis. The preparation method of the $Ag_H$-S-$Al_2O_3$-$Ni^{2+}$ and S-$Al_2O_3$-$Ni^{2+}$ catalysts can be found in our previous report.[8]

**Table S6.** Catalytic performance of the $Ag_H$-S-$Al_2O_3$-$Ni^{2+}$ catalyst for the cleavage of *α*-O-4 and *β*-O-4 model compounds.

| Entry | Catalysts | Reactant | Condition | Conversion |
|---|---|---|---|---|
| 1 | $Ag_H$-S-$Al_2O_3$-$Ni^{2+}$ | 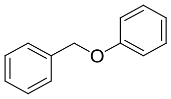 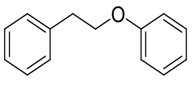 | 90°C, 440 nm LED, 0.5 W $cm^{-2}$, 20 h of reaction time | 86% (*α*-O-4) 96% (*β*-O-4) |

Reaction conditions: reactant (0.05 mmol), KOH (0.15 mmol), 2 mL of IPA solvent, 1 atm argon atmosphere, 10 mg of catalyst. The preparation method of the $Ag_H$-S-$Al_2O_3$-$Ni^{2+}$ catalyst can be found in the reference.[8]

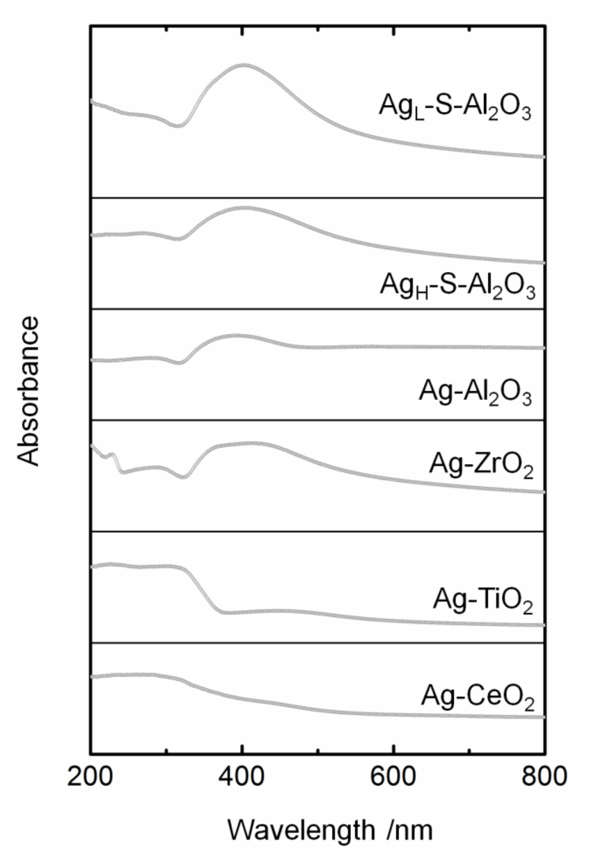


**Figure S1.** DR UV-Vis spectra of the supported Ag NPs.

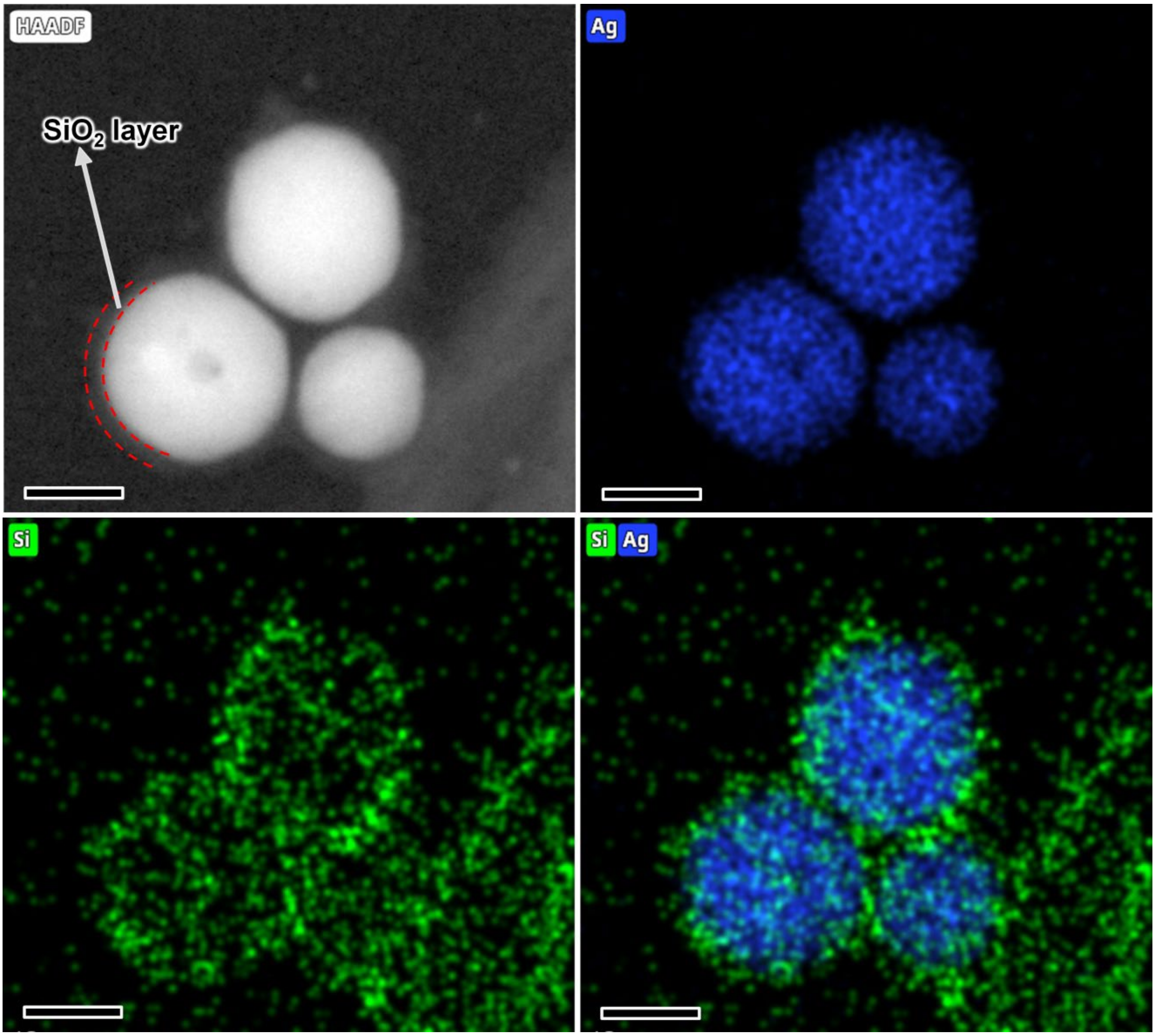


**Figure S2.** STEM image and elemental mapping of the silica-coated $Ag_H$-S-$Al_2O_3$ catalyst. The scale bars in the images are 10 nm.

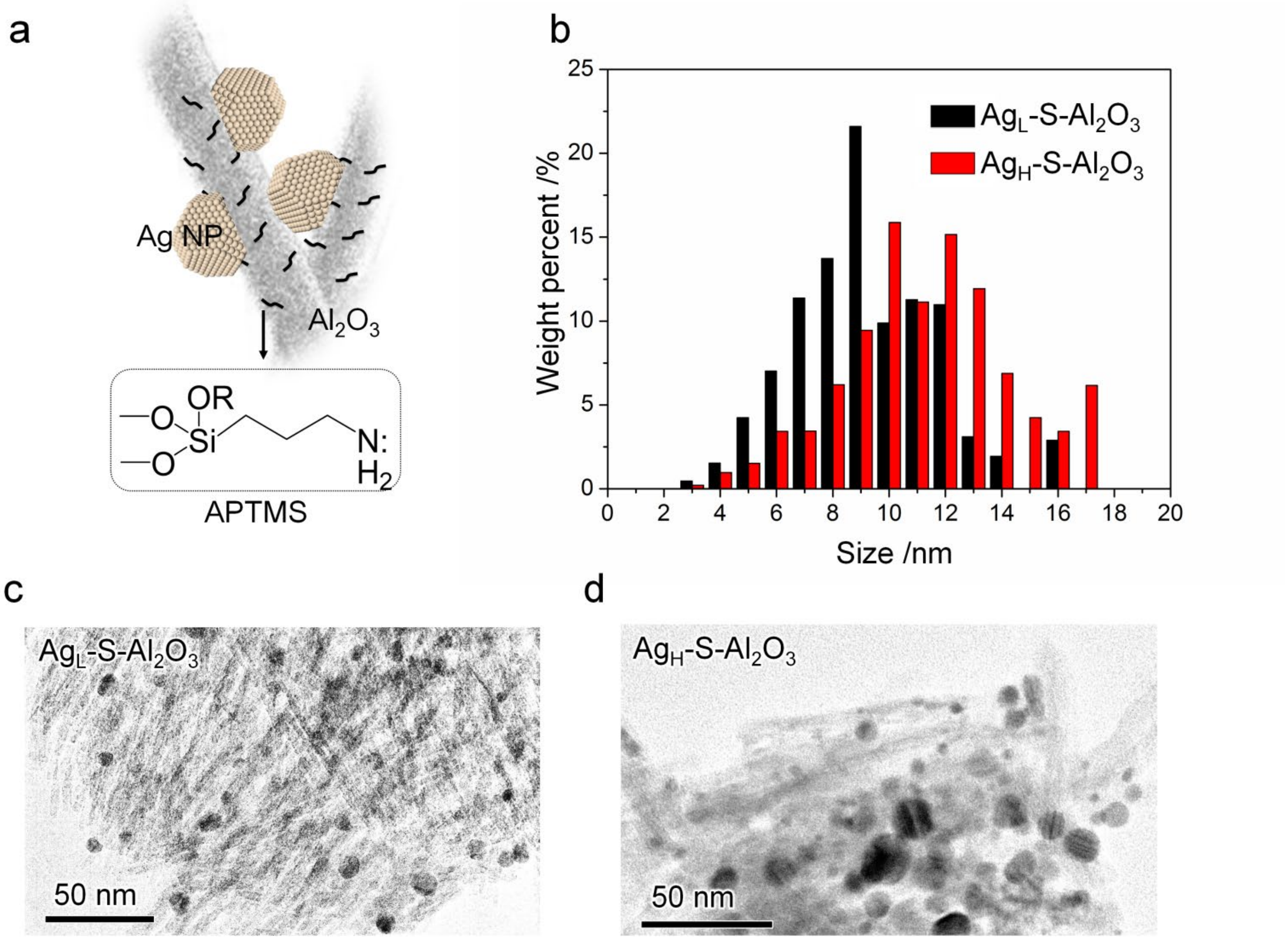


**Figure S3.** (a) Schematic drawing of $Ag_H$-S-$Al_2O_3$ catalyst. (b) Ag NP Size distributions of $Ag_H$-S-$Al_2O_3$ and $Ag_L$-S-$Al_2O_3$ catalysts. (c) and (d) Typical TEM images of $Ag_L$-S-$Al_2O_3$ and $Ag_H$-S-$Al_2O_3$ catalysts, respectively.

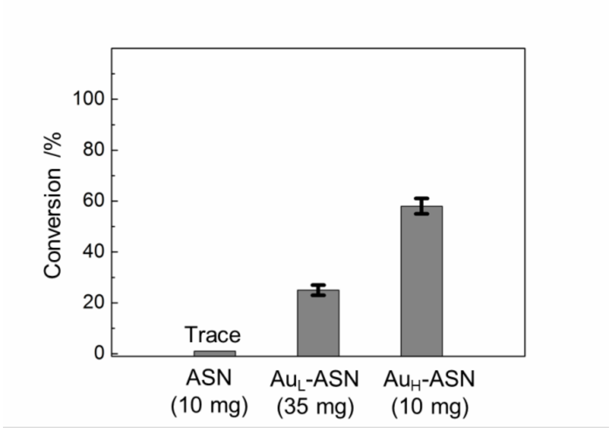


**Figure S4**. Catalytic performances of the photocatalysts containing Au NPs for C-O bond cleavage of the *β*-O-4 model compound under visible light irradiation and in the dark. Reaction conditions: *β*-O-4 model (0.05 mmol), KOH (0.15 mmol), 2 mL of isopropanol (IPA) solvent, 1 atm argon atmosphere, 525 nm wavelength light at 0.5 W $cm^{-2}$ of intensity, 20 h of reaction time at 90°C.

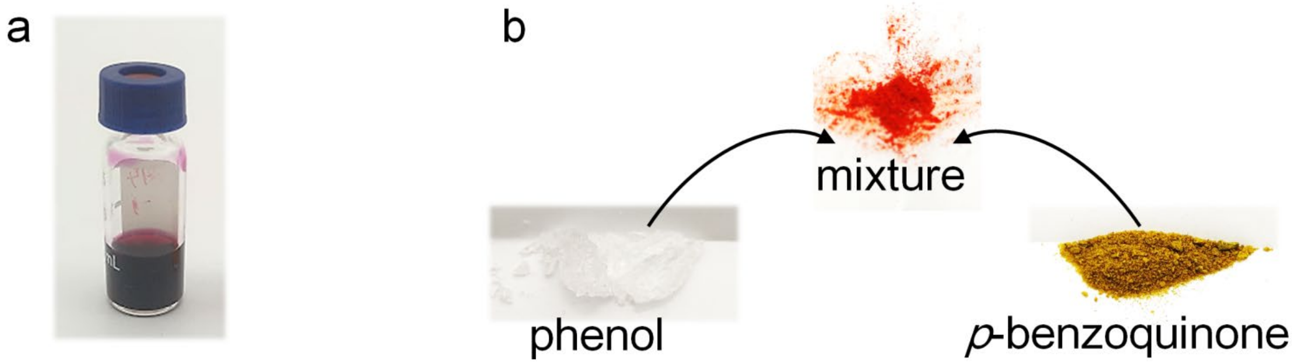


**Figure S5**. (a) The photograph of the product obtained using methanol as the solvent. Reaction conditions: 1-phenoxy-2-phenylethane (*β*-O-4 model, 0.05 mmol), KOH (0.15 mmol), 2 mL of methanol solvent, 1 atm Ar atmosphere, 10 mg of $Ag_H$-S-$Al_2O_3$ catalyst, 440 nm wavelength light: 0.5 W $cm^{-2}$, 20 h of reaction time at 90$^o$C. (b) The red mixture can be obtained by grinding *p*-benzoquinone with phenol.

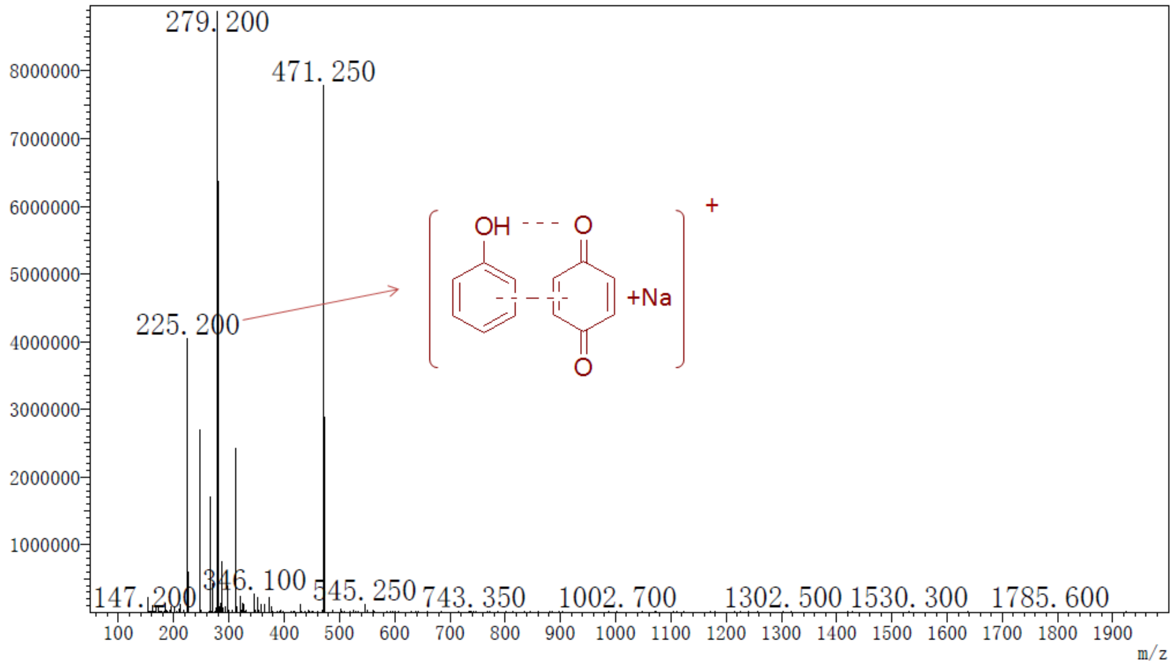


**Figure S6.** Detection of the product obtained in the 6th column of Figure 2c by LC-MS. m/z (ESI) calculated for ($C_{17}H_{24}O_3$) $[M+Na]^+$: 225.05, found: 225.200.

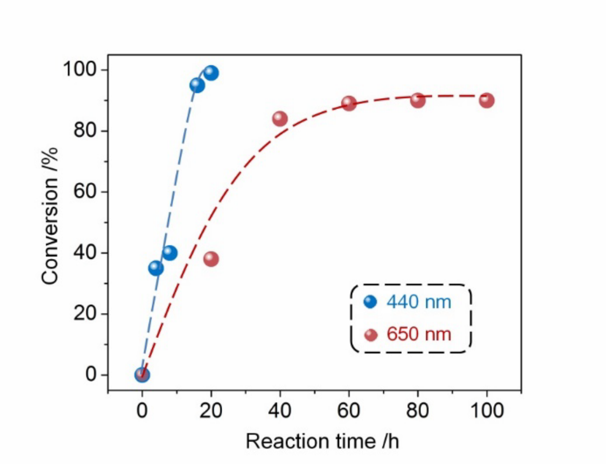


**Figure S7** The time course study of 1-phenoxy-2-phenylethane ($\beta$-O-4 model) using $Ag_H$-S-$Al_2O_3$ catalyst under irradiation at the same intensity (0.5 W $cm^{-2}$) with different wavelengths. Reaction conditions are the same as those in Figure 2a.

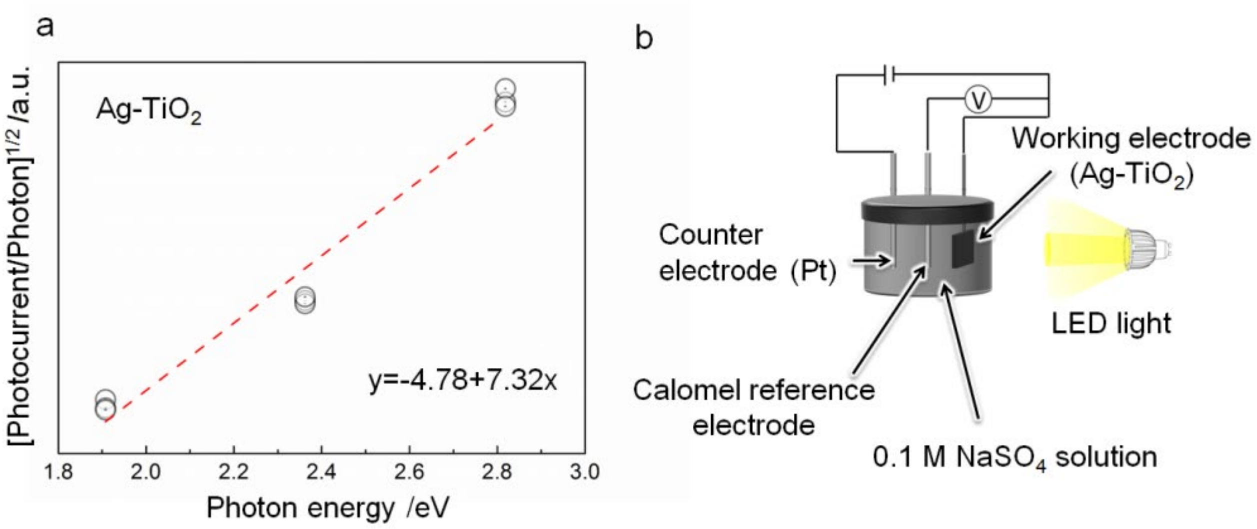


**Figure S8.** (a) Square root of the photocurrent of Ag-$TiO_2$ catalyst as a function of incident photon energy. The photocurrent values were calculated from Figure 4a and normalised to the incident photon flux. (b) The setup of the three-electrode system with different wavelength LEDs as the light sources, in which the light intensity was 0.1 W·cm$^{-2}$.

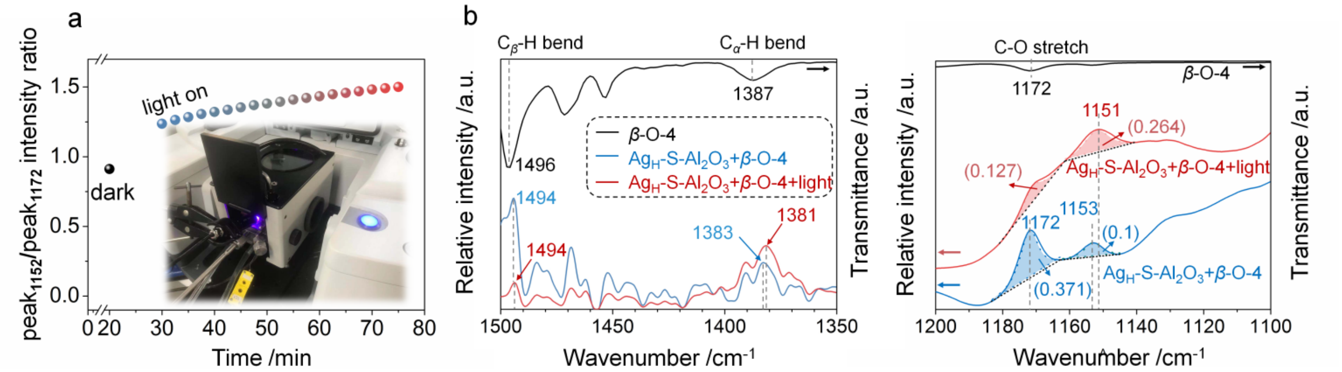


**Figure S9** (a) The irradiation time dependence of intensity ratios of the two typical peaks (~1152 and 1172 $cm^{-1}$) was calculated from Figure 5. The inset shows the photograph of the experimental setup. The reactant-catalyst mixture was dispersed in KBr (2% concentration) and placed in a DRIFTS cell. Before the test, the sample was purged with Ar flow for 10 min and then sealed. The sample was heated at 90°C, and a 405 nm wavelength light at 0.5 W $cm^{-2}$ of intensity was used to acquire in situ spectra continuously. (b) FTIR spectra of *β*-O-4 compound (black), in situ DRIFT spectra of *β*-O-4 compound adsorbed on $Ag_H$-S-$Al_2O_3$ catalyst collected in the dark (blue) and under 405 nm light irradiation (red).

To understand how reactant chemisorption on the catalyst and light irradiation influence the relevant, we compared the infrared absorption locations of the bonds under different conditions. As shown in the left panel of Figure S9b, the peaks at about 1496 and 1387 $cm^{-1}$ can be assigned to the bending mode of $C_\beta$-H and $C_\alpha$-H bonds, respectively. Shifts to lower wavenumbers can be observed on the adsorbed *β*-O-4 compound and the adsorbed reactant under irradiation, indicating the bonds are elongated by chemisorption and light-induced electron transfer. Importantly, compared with $C_\beta$-H, the redshifts of $C_\alpha$-H bond are more obvious. This corroborates that Ag NPs can specifically activate a $C_\alpha(sp^3)$-H bond under irradiation. Besides, as displayed in the right panel of Figure S9b, the peaks at about 1172 $cm^{-1}$ and 1152 $cm^{-1}$ can be ascribed to the characteristic C-O bond stretch mode of the free and chemisorbed ether on the catalyst, respectively. The area of characteristic peaks were integrated and their ratios were compared to avoid a bias analysis due to baseline shift. A similar shift to lower wavenumbers as C-H bond can be observed. It can be found that the area ratio of the 1152/1172 $cm^{-1}$ peaks of *β*-O-4 compound adsorbed on $Ag_H$-S-$Al_2O_3$ catalyst increased obviously under illumination (from ~0.27 to ~2.1). This indicates that more chemisobed *β*-O-4 compound with elongated C-O bond can be observed on the catalyst under irradiation, suggesting that the illumination facilitates chemisorption of the *β*-O-4 compound and bond cleavage.

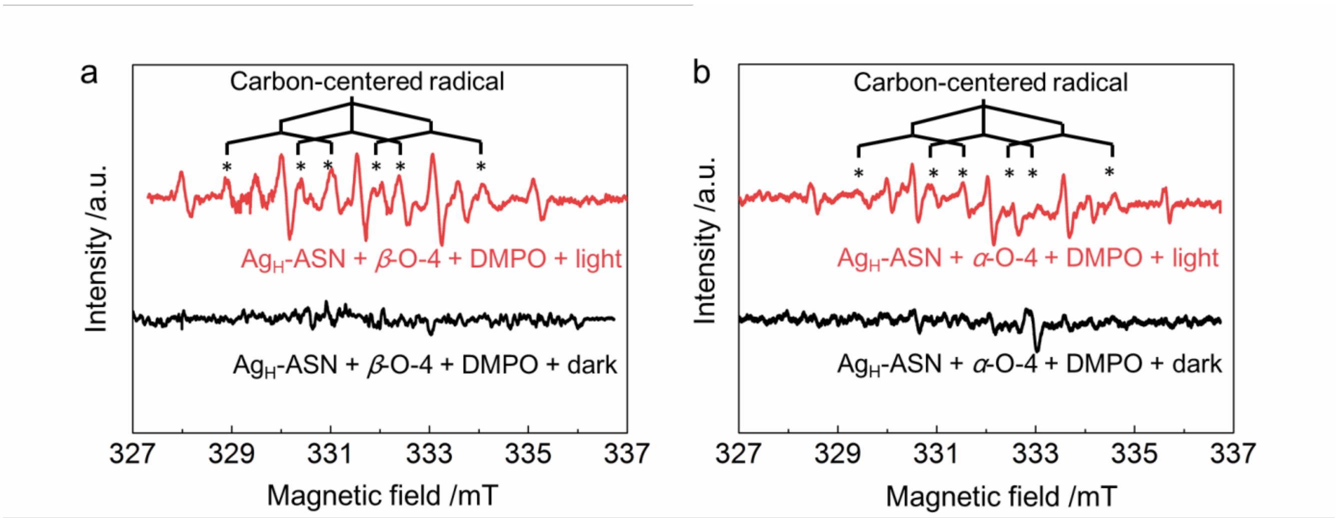


**Figure S10.** Comparison of DMPO spin-trapping EPR spectra under light irradiation and in the dark. The six-line spectra (signed with (*)) are the characteristic signals of DMPO-•$C_{14}H_{13}O$ (a) and DMPO-•$C_{13}H_{12}O$ (b), respectively. These EPR peaks over the $Ag_H$-S-$Al_2O_3$ catalyst emerged after the irradiation and can be assigned to carbon-centred radicals.[9] Reaction conditions: lignin model compounds (0.05 mmol), DMPO (0.15 mmol), KOH (0.15 mmol), 10 mg of $Ag_H$-S-$Al_2O_3$ catalyst, 2 mL of IPA solvent, 4 h of reaction time at 90°C, the light source was a LED chip (440±5 nm) at 0.5 W $cm^{-2}$.

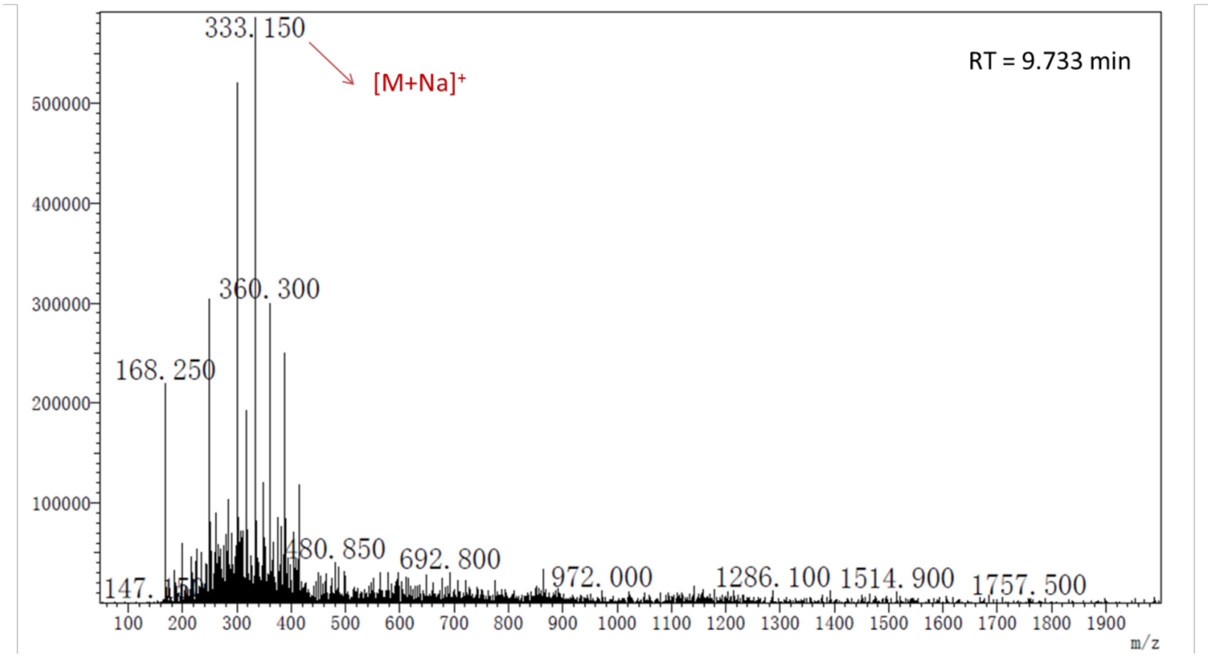


**Figure S11.** Detection of aryl radical trap product by LC-MS. Reaction conditions: *β*-O-4 model (0.05 mmol), KOH (0.15 mmol), 10 mg of $Ag_H$-S-$Al_2O_3$ catalyst, 2 mL of IPA solvent, 4 h of reaction time at 90°C, the light source was a LED chip (440±5 nm) at 0.5 W $cm^{-2}$. m/z (ESI) calculated for ($C_{20}H_{24}NO_2$) $[M+Na]^+$: 333.17, found: 333.150.

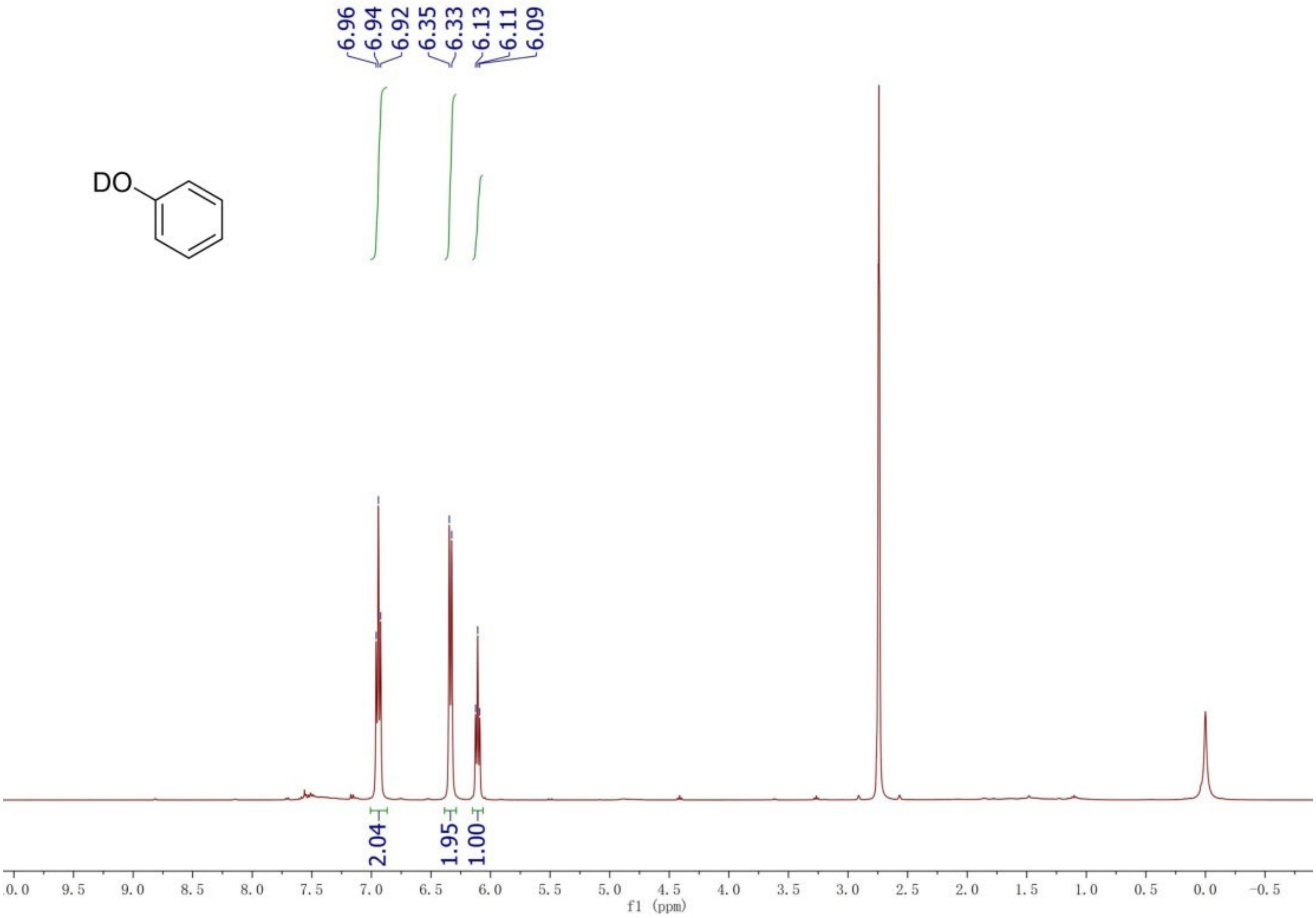


**Figure S12.** $^{1}H$ NMR spectrum of phenol produced in the cleavage of the *β*-O-4 compound by using $Ag_H$-S-$Al_2O_3$ catalyst in IPA-d8 solvent under irradiation. $^{1}H$ NMR (400 MHz, DMSO-$d_6$) δ 6.96-6.92 (m, 2H), 6.34 (d, $J$ = 8.0 Hz, 2H), 6.13-6.09 (m, 1H). The product was filtered through a filter (pore size 0.22 μm) to remove the catalysts and dried in a vacuum oven at 60°C to remove the solvent before the analysis.

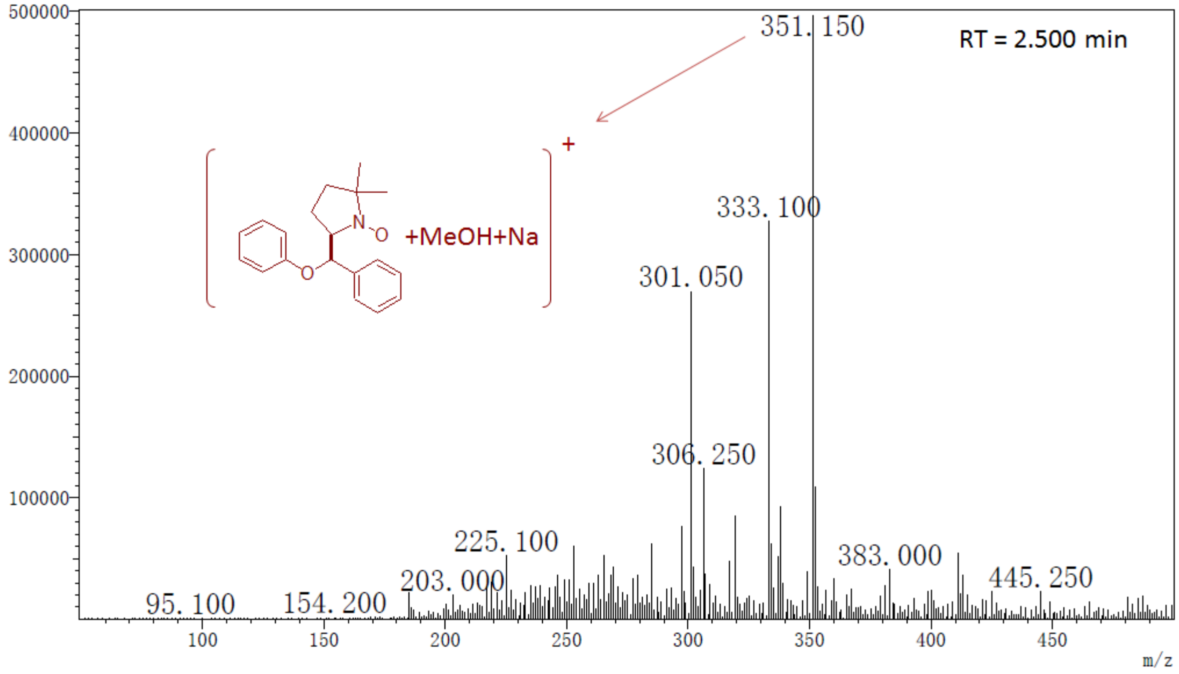


**Figure S13.** Detection of aryl radical trap product by LC-MS. Reaction conditions: *α*-O-4 model (0.05 mmol), KOH (0.15 mmol), DMPO (0.15 mmol), 5 mg of $Ag_H$-S-$Al_2O_3$ catalyst, 2 mL of IPA solvent, 4 h of reaction time at 90°C, the light source was a LED chip (440±5 nm) at 0.5 W $cm^{-2}$. m/z (ESI) calculated for ($C_{19}H_{22}NO_2$) $[M+MeOH+Na]^+$: 351.18, found: 351.15.

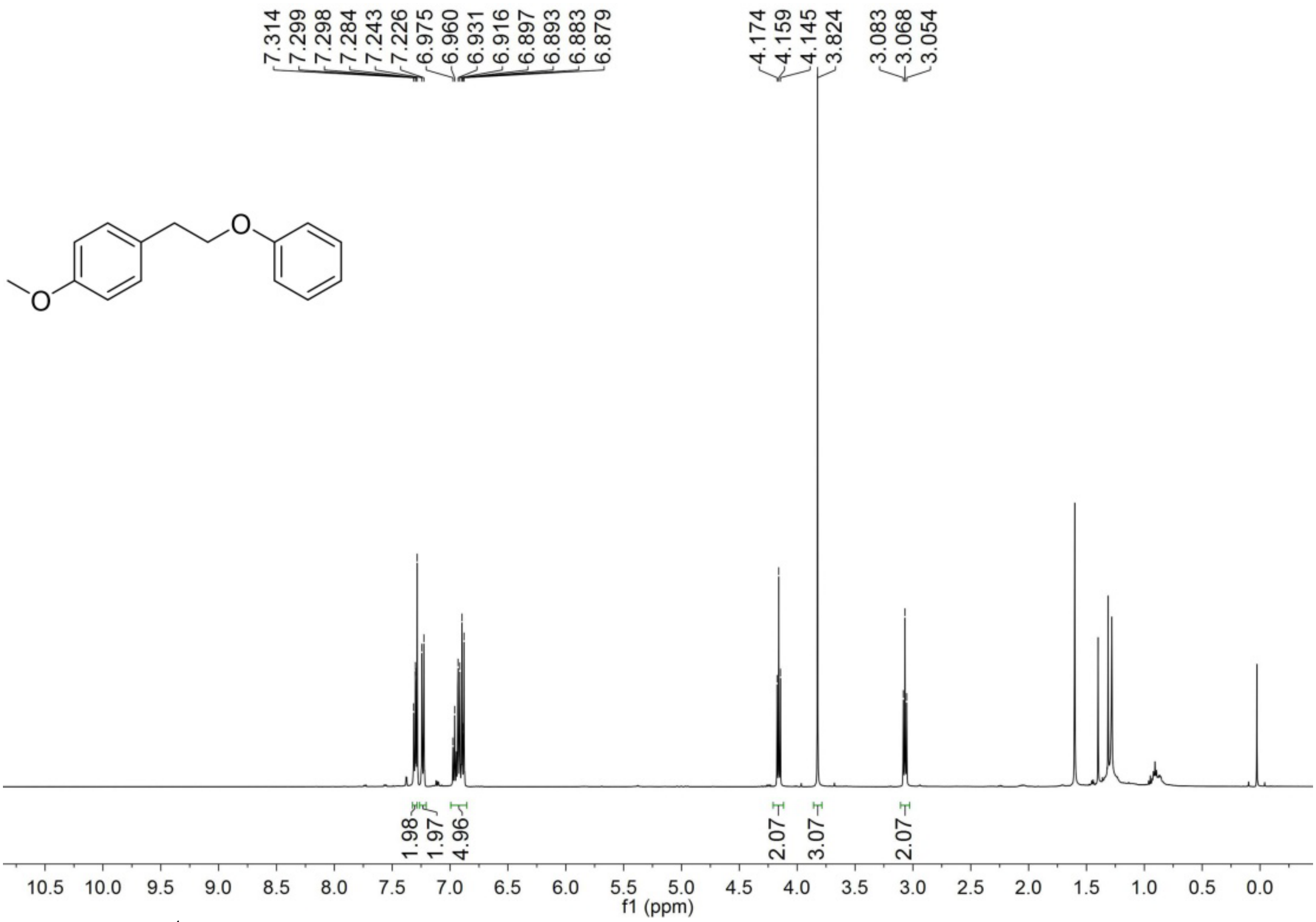


**Figure S14.** $^1H$ NMR spectrum of the purchased 1-methoxy-4-(2-phenoxyethyl)benzene from Foshan Haer Biotechnology Co., Ltd. $^1H$ NMR (500 MHz, $CDCl_3$) δ 7.31-7.30 (m, 2H), 7.23 (d, *J* = 8.5 Hz, 2H), 6.98-6.88 (m, 5H), 4.17-4.15 (m, 2H), 3.82 (s, 3H), 3.08-3.05 (m, 2H), 1.60 (s, 2H).

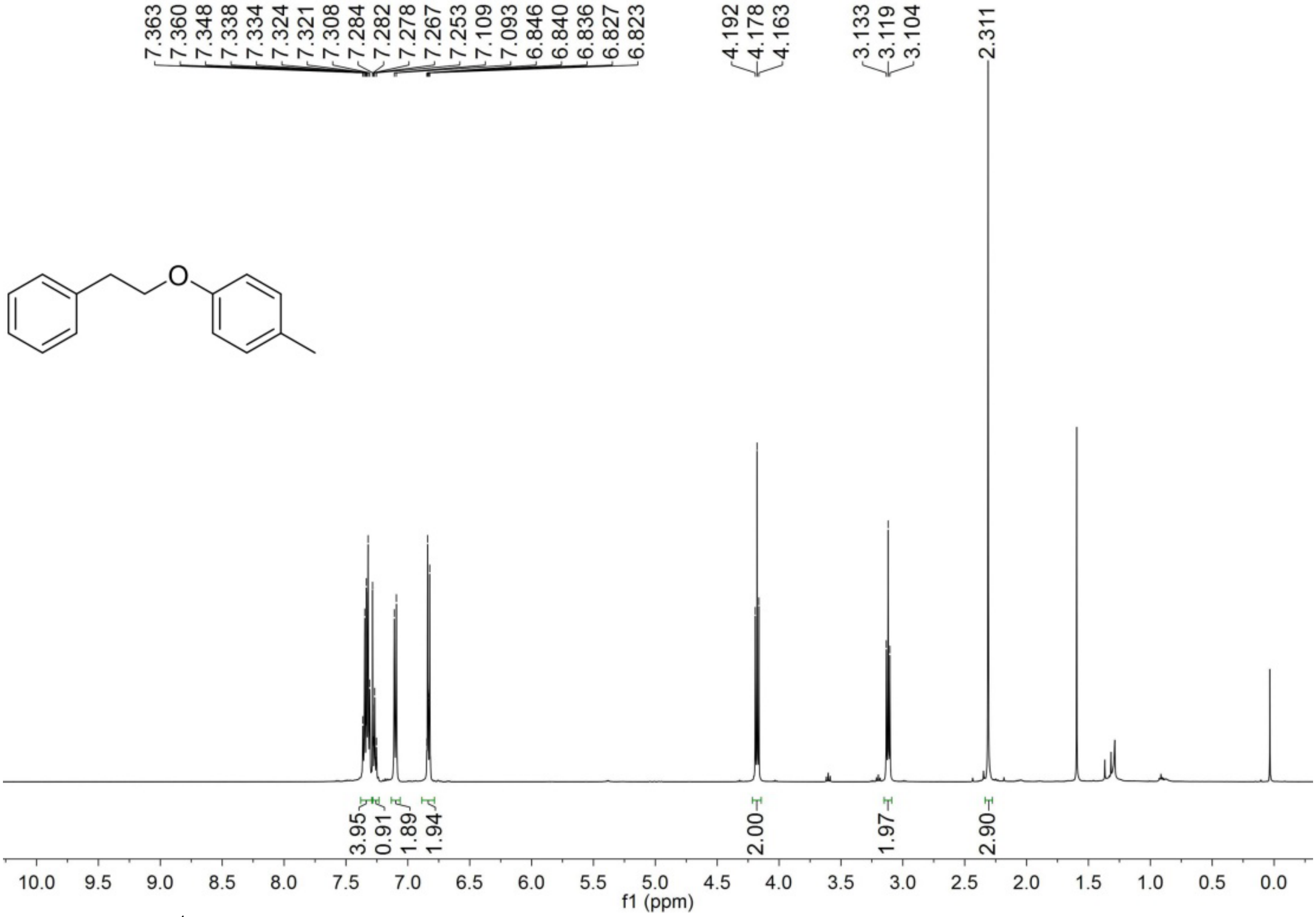


**Figure S15.** $^{1}$H NMR spectrum of the purchased 1-methyl-4-(2-phenylethoxy)benzene from Foshan Haer Biotechnology Co., Ltd. $^{1}$H NMR (500 MHz, $CDCl_3$) δ 7.36-7.31 (m, 4H), 7.28-7.25 (m, 1H), 7.10 (d, *J* = 8.0 Hz, 2H), 6.85-6.82 (m, 2H), 4.19-4.16 (m, 2H), 3.13-3.10 (m, 2H), 2.31 (s, 3H), 1.60 (s, 1H).

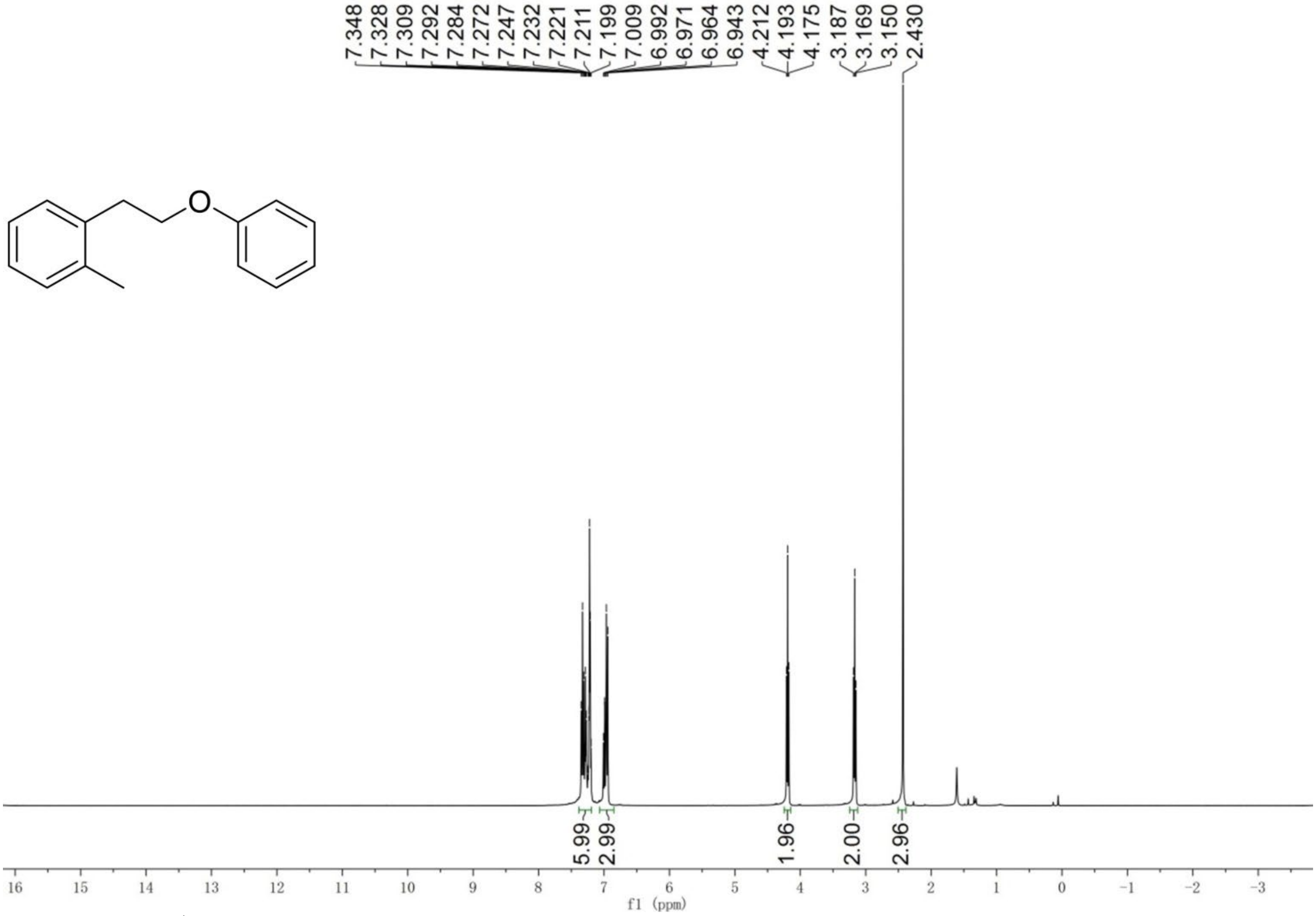


**Figure S16.** $^1$H NMR spectrum of 1-methyl-2-(2-phenylethoxy)benzene provided by Prof. Xueqiang Wang from Hunan University. $^1$H NMR (400 MHz, $CDCl_3$) δ7.35-7.20 (m, 6H), 7.01-6.94 (m, 3H), 4.21-4.18 (m, 2H), 3.19-3.15 (m, 2H), 2.43 (s, 3H).

**Author Contributions**

Pengfei Han performed all the experiments and developed the mechanism with Huai-Yong Zhu. Jianfeng Jia conducted the BDE calculation. Xin Mao and Aijun Du calculated activation energies. Yichao Jin conducted the in situ DRIFT analysis. Eric R. Waclawik and Jin-Cai Zhao provided valuable discussion and revised the manuscript with significant improvements. Huai-Yong Zhu and Sarina Sarina supervised the project. The manuscript was written through the contributions of all authors.

**References:**


[1] H. Y. Zhu, J. D. Riches, J. C. Barry, *Chem. Mater.* **2002**, *14*, 2086-2093.

[2] J. F. Li, X. D. Tian, S. B. Li, J. R. Anema, Z. L. Yang, Y. Ding, Y. F. Wu, Y. M. Zeng, Q. Z. Chen, B. Ren, Z. L. Wang, Z. Q. Tian, *Nat. Protoc.* **2013**, *8*, 52-65.

[3] a) G. Kresse, J. Furthmüller, *Comput. Mater. Sci.* **1996**, *6*, 15-50; b) G. Kresse, J. Furthmüller, *Phys. Rev. B* **1996**, *54*, 11169.

[4] a) P. E. Blöchl, *Phys. Rev. B* **1994**, *50*, 17953; b) G. Kresse, D. Joubert, *Phys. Rev. B* **1999**, *59*, 1758.

[5] J. P. Perdew, K. Burke, M. Ernzerhof, *Phys. Rev. Lett.* **1996**, *77*, 3865.

[6] M. W. Jarvis, J. W. Daily, H.-H. Carstensen, A. M. Dean, S. Sharma, D. C. Dayton, D. J. Robichaud, M. R. Nimlos, *J. Phys. Chem. A* **2011**, *115*, 428-438.

[7] C. Reichardt, T. Welton, Appendix A. Properties, Purification, and Use of Organic Solvents. *In solvents and solvent effects in organic chemistry;* Weinheim: Wiley-VCH, **2010**.

[8] P. Han, T. Tana, Q. Xiao, S. Sarina, E. R. Waclawik, D. E. Gómez, H. Y. Zhu, *Chem* **2019,** *5*, 2879-2899.

[9] a) F. Chen, Y. Xie, J. He, J. Zhao, *J. Photochem. Photobiol. A,* **2001**, *138*, 139-146; b) H. Li, F. Qin, Z. Yang, X. Cui, J. Wang, L. Zhang, *J. Am. Chem. Soc.* **2017**, *139*, 3513-3521.
